# Cognition in the cockpit: assessing instructional modalities in pilot training simulations

**DOI:** 10.3389/fpsyg.2025.1625321

**Published:** 2025-10-27

**Authors:** Laurie-Jade Rochon, Alexander John Karran, Thadde Rolon-Merette, François Courtemanche, Constantinos Coursaris, Sylvain Senecal, Pierre-Majorique Léger

**Affiliations:** Tech3lab, HEC Montreal, Department of Information Technologies, Montreal, QC, Canada

**Keywords:** pilot training, instructional modality, cognitive load, visual attention, motivation, human computer interface, cognition

## Abstract

**Introduction:**

Flight Simulators (FS) play a critical role in pilot training, yet the increasing use of automated modules in FS raises questions about how instructional delivery methods influence learning. This study investigates how different FS instruction modalities affect student pilots’ cognitive states and performance.

**Methods:**

A between-subjects experiment was conducted with 30 flight-school students using Microsoft Flight Simulator 2020 under Visual Flight Rules (VFR). Participants were randomly assigned to one of three instruction modalities: audio-only, text-only, or combined audio-text. Each participant completed two tasks: (1) an instructional flight with guided instructions and (2) a solo evaluation flight without guidance. Measures included visual transition entropy (to assess visual scanning), emotional valence, cognitive load, motivation, and flight performance metrics.

**Results:**

During the evaluation flight, the text-only and combined audio-text groups showed significantly lower visual transition entropy, indicating more organized visual scanning. The text-only group also exhibited higher emotional valence, reflecting greater motivation and engagement. No significant differences were found in overall flight performance or cognitive load, although trends suggested higher perceived immersion and motivation in the text-only condition.

**Discussion:**

Textual instructional delivery appears to support more efficient visual scanning and greater engagement, aligning with the Cognitive Theory of Multimedia Learning while highlighting its boundary conditions in aviation contexts. Although performance metrics were unaffected in this short session, textual information may be advantageous for specific flight segments and scenarios lacking live instruction. Further research should examine longer or repeated training sessions.

## 1 Introduction

Advances in aviation training are helping to reshape how pilots prepare for flight and maintain expertise and readiness. Flight simulators (FS) offer a controlled, risk-free environment for skill acquisition, increasingly incorporating automated instruction. Understanding how instructional modalities affect trainee pilots’ cognitive and affective learning is crucial. While live instruction remains the gold standard, the shift toward self-directed FS training requires careful design evaluation. This study examines how sensory modalities in FS instruction influence cognitive states, visual strategies, and performance, aiming to inform evidence-based training improvements.

Flight simulators, certified by aviation authorities such as Transport Canada, are essential for pilot training, reducing costs and risks (Aragon and Hearst, 2005). Recent advancements have made FS more affordable, powerful, and versatile, enabling independent use without live instructors. Uncertified platforms such as Microsoft Flight Simulator 2020 (MFS) (Xbox Game Studios, 2020) allow users to explore global maps, familiarize themselves with avionics and procedures, and practice maneuvers across different scenarios (Callender et al., 2009). Simulator training typically involves three components: a simulator, a structured syllabus, and an instructor (Myers et al., 2018). While FS training is instructor-led conventionally, modern systems integrate training syllabi and virtual instructions via flight objectives and visual guides. However, research indicates instructors have a more significant impact on student progress than syllabus or simulator variations (Valverde, 1973). This research highlights a need to evaluate FS instructional methods and their interaction with FS fidelity, efficiency, and learning outcomes.

A significant challenge in FS instructional design is developing content that aligns with human information processing abilities and mechanisms while minimizing interference with aircraft operations. The human information processing system governs how individuals perceive, interpret, and store information (Schneider and Shiffrin, 1977). Effective learning depends on managing cognitive resources like working memory and attention, given that overload impairs performance, particularly in high-demand environments. However, overly simplistic content may fail to engage learners, reducing cognitive processing (Fredricks et al., 2004). Thus, instructional design must balance information load to enhance understanding and knowledge assimilation.

Given these factors, we can infer that a critical design element for FS training is the modality of information, i.e., the type and amount of visuospatial and auditory information presented. Content can be unimodal (delivered via a single sensory channel, visual or auditory) or bimodal (integrating both, e.g., on-screen animations with narration). Research related to how sensory modalities impact FS learning is limited. In this regard, the Cognitive Theory of Multimedia Learning (CLT) suggests that presenting all learning content visually can overload visual working memory due to competing cognitive demands (Baddeley, 1992; Mayer, 2024). Conversely, the modality effect posits that distributing information across auditory and visual channels may reduce cognitive load and enhance learning (Mayer and Pilegard, 2005). Thus, the extent to which FS instructional modalities influence pilot trainees’ learning and cognitive states remains unclear.

To determine the extent to which instructional modalities influence pilot trainees’ learning and cognitive states, we conducted a laboratory study involving 30 flight-school pilots. Participants completed two *visual flight rules* (VFR) flights in the MFS: one VFR flight with a virtual instructor and one VFR solo flight (i.e., without an instructor). Participants were divided into three experimental groups where the sensory modality of flight instructions was manipulated: one-third of the participants were presented with *bimodal* (audio and text) flight instructions, a third of the participants were presented with *unimodal-audio* flight instructions only, and the remaining participants were presented with *unimodal-text* flight instructions. The learning performance and cognitive states were assessed using psychometric instruments and physiological tools during the VFR flights. To the best of our knowledge, this study is, to our knowledge, the first to determine how the sensory modality of FS instructions affects pilots’ cognitive learning states and learning performance within an educational, ecologically valid, and widely used FS and training context. In the following sections of this manuscript, we present the background and theoretical framework, methods, measures, statistical analysis, and then a discussion and conclusion of the results in context.

## 2 Background and theoretical framework

In this section, a summary of the extant literature in the Human-Computer Interface (HCI) and psychology fields related to the manipulation of navigation instruction sensory modalities is presented, followed by the theoretical framework that serves as a foundation for the current study and posit a number of hypotheses that seek to answer our motivating research question “*To what extent do information modalities affect trainee pilot cognitive states and performance*.”

### 2.1 Multimedia learning: the “modality principle”

The Cognitive Theory of Multimedia Learning (CTML) (Mayer and Pilegard, 2005) posits that integrating text and images enhances learning more than text alone (Butcher, 2014). CTML is based on three core assumptions: dual-channel processing (Baddeley, 1992; Paivio, 1991), active construction of mental models from verbal and visual inputs (Mayer and Mayer, 2005; Wittrock, 1989), and the limited capacity of each processing channel (Baddeley, 1992). The brain processes information through distinct pathways, such as visuospatial and auditory channels, each engaging different neural substrates (Baldwin et al., 2012). Expanding on CTML, Moreno’s Cognitive-Affective Theory of Learning (CATL) highlights the role of motivation and metacognition in learning. It suggests that affective and metacognitive factors enhance engagement and regulate cognitive processes (Moreno, 2006). Research stemming from CTML and CATL has demonstrated that multiple factors, such as modality, segmentation, and pre-training affect cognitive load and learning, underlining that not all multimedia applications are equally effective (Noetel et al., 2022).

These insights informed several multimedia design principles, notably the modality principle, which states that learning improves when verbal information is delivered through narration rather than on-screen text. This approach reduces cognitive load by distributing processing between auditory and visual channels (Mayer and Pilegard, 2005). Extensively studied across fields like geometry, biology, and virtual reality (Moreno, 2006; Jeung et al., 1997), findings consistently show superior learning outcomes when speech replaces text, regardless of media characteristics (Moreno, 2006). However, its application in flight simulation (FS) remains underexplored. FS training involves high-element interactivity with complex stimuli, requiring learners to simultaneously process instructional content, environmental cues, and psychomotor tasks (Pociask and Morrison, 2004). This complexity may modulate the modality principle’s effectiveness due to the heightened cognitive demands of FS training.

To our knowledge, no studies have directly compared the learning effect of different sensory modalities in flight simulator (FS) training. Prior FS research on the modality principle has primarily focused on data link studies, which examine auditory versus textual communication, including a redundant condition combining both (Lancaster and Casali, 2008; Rehmann, 1997; Latorella, 1998; Helleberg and Wickens, 2003; McGann et al., 1998). Data links transmit digital flight information between aircraft operators and air traffic controllers (ATC), often used in scenarios where radio communication is impractical, such as oceanic crossings (Latorella, 1998). These studies assess the benefits of text-based versus voice-based ATC communications in multitasking environments similar to those in the present study.

Research suggests that both auditory and textual instructions enhance performance in aircraft operations, each with distinct trade-offs. Textual instructions offer permanence and allow for accuracy verification, making them effective for spatial tasks when paired with manual responses, as described by the stimulus-central processing-response (SCR) compatibility model (Rehmann, 1997; Wickens et al., 2021). However, they can increase response times and cognitive load compared to auditory instructions (Lancaster and Casali, 2008). Auditory instructions provide advantages such as pre-emption effects, heightened urgency, and better retention, making them particularly effective for clarifying navigation messages (Latorella, 1998; Helleberg and Wickens, 2003). Moreover, they prevent conflicts associated with translating text into spatial relationships (Brooks, 1968) but may disrupt ongoing visual tasks by diverting attention (Latorella, 1998; Wickens and Liu, 1988). Thus, a bimodal condition, integrating auditory and textual instructions, may improve execution accuracy through redundancy, enabling cross-verification of information. However, this potentially comes at the cost of efficiency, as it increases response times (Lancaster and Casali, 2008).

### 2.2 Monitoring pilots’ cognitive, attentional, and emotional learning states

**Figure 1:**
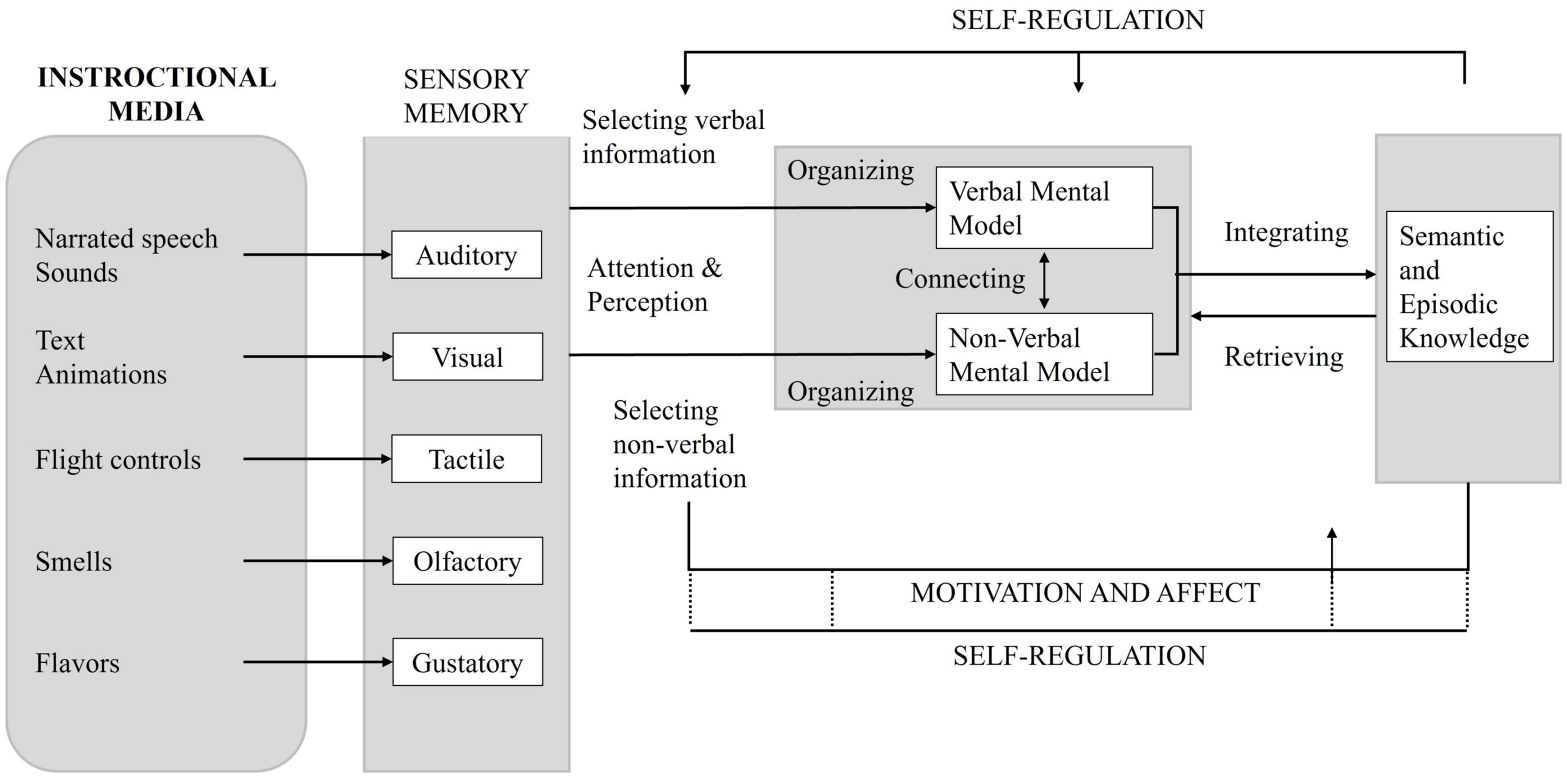
Cognitive-affective theory of learning with media, adapted from Mayer (2005).

Based on the CTML (Mayer and Mayer, 2005) and the CATL (Moreno, 2006; Moreno, 2005; Moreno, 2007; Moreno, 2009), this study proposes a framework, shown in Figure 1, to evaluate the subjects’ learning experience that enhances the cognitive perspective by taking perceptual, attentional, motivational, and affective aspects into account. As such, the modality conditions can be treated as features of instructional media that shape three interrelated components of learning: attentional processes (selection and monitoring of relevant information), cognitive load (the effort required to organize and process information), and affective–motivational processes (e.g., immersion, interest, and self-regulation). Therefore, it is crucial to examine whether the modalities differentially influence these components during instruction and whether variation in these components is associated with subsequent learning and transfer. In this way, CATL serves as the organizing lens that links media to cognitive–affective processes and, ultimately, to learning outcomes.

### 2.3 Cognitive load

Pilots must continuously monitor critical instrument panel cues to operate an aircraft safely and efficiently. Processing these signals and generating appropriate psychomotor responses impose a substantial cognitive load, defined as the working memory resources required for a task (Kalyuga, 2008). In multimedia learning, cognitive load increases when learners must expend additional effort to integrate information from multiple sensory modalities, diverting resources from actual learning. Effective instructional design helps to mitigate this by reducing extraneous cognitive processing (Mayer, 2014).

Cognitive load can be assessed through perceptual indices, such as verbal reports, psychometric instruments, and psychophysiological markers (Charlton, 2002). The most widely used physiological index is pupil dilation, which occurs spontaneously and involuntarily, making it a non-invasive measure (van der Wel and van Steenbergen, 2018; Laeng and Alnaes, 2019). Pupillary light reflexes produce large changes (several millimeters), whereas cognitive activity induces smaller fluctuations (0.1–0.5 mm) (Beatty, 1982). Studies have consistently shown that pupil dilation increases with cognitive demand, making it a reliable indicator of cognitive load. Early research analyzed raw pupil diameter data (Hamel, 1974; Nunnally et al., 1967; Scott et al., 1967), but individual differences in baseline pupil size limited comparability. Contemporary studies employ transformation methods to standardize pupillary responses (Beatty, 1982; Attard-Johnson et al., 2019; Weber et al., 2021), notably the Percentage Change in Pupil Diameter (PCPD). PCPD is calculated as the difference between task-related pupil diameter and a pre-stimulus baseline, divided by the baseline. This baseline typically represents the average pupil diameter over a few seconds before task onset (Attard-Johnson et al., 2019).

### 2.4 Visual attention

Visual attention is essential for pilot learning. It requires the division of focus across multiple tasks, including cockpit monitoring, flight instruction processing, and external scanning (OTW), all of which impose high attentional demands. As pilots train, they develop efficient attention allocation strategies, balancing these tasks for safe and effective operation. Differences between novice and expert pilots highlight the importance of this skill: novices focus narrowly on cockpit instruments, whereas experts integrate external cues, enhancing situational awareness and decision-making.

Eye tracking is a powerful tool for assessing attentional processes. Pilot training research employs various eye movement metrics, including fixations, saccades, and Areas of Interest (AOIs), to evaluate cognitive, perceptual, and attentional states (Glaholt, 2014). From these data, multiple metrics can be derived to infer visual attention. For instance, gaze transition entropy (GTE) quantifies gaze pattern randomness or complexity, with higher values indicating more frequent shifts between AOIs. GTE is defined by Equation 1:


(1) $$H(x)=-\sum_{i=1}^{n}p_{i}\sum_{j=1}^{n}p(i,j)\log_{2}p(i,j)$$

where *i* represents the “from” AOI, *j* represents the “to” AOI, *p_i_* represents the stationary distribution, and *p*_(*i*,*j*)_ represents the probability of transitioning from *i* to *j*. Higher GTE denotes more randomness and more frequent switching between AOIs. Typically, GTE is normalized by calculating the ratio of GTE to H_max_, which represents the maximum theoretical entropy to account for the number of AOIs, which is calculated by Equation 2:


(2) $$H_{\max}=\log_{2}(\text{Number of AOIs})$$

This normalization ensures that GTE/Hmax reflects the relative complexity of gaze patterns regardless of the number of AOIs, allowing for standardized comparisons across tasks and conditions. GTE is influenced by both intrinsic and extrinsic factors. For instance, higher task cognitive load (Van De Merwe et al., 2012; Ephrath et al., 1980; van Dijk et al., 2011) and levels of stress (Allsop and Gray, 2014) correlated with higher GTE while task complexity. Moreover, recent findings indicate that task complexity reduces GTE (Diaz-Piedra et al., 2019), while expertise tends to increase it under comparable task conditions (Lounis et al., 2021).

Another metric which is often is the ambient-focal K coefficient, introduced by Krejtz et al. (2016), as it captures changes in visual scanning behavior throughout a task. The coefficient can be obtained using Equation 3. Negative and positive ordinates of *K* indicate ambient viewing (governing initial scene exploration) and focal viewing (common during scene inspection), respectively. *K* is derived as the mean difference between standardized values (z-scores) of each saccade amplitude (*a_i + 1_*) and its preceding *i^th^* fixation duration (*d_i_*):


(3) $$K_{i}=\frac{d_{i}-\mu_{d}}{\sigma_{d}}-\frac{a_{i+1}-\mu_{a}}{\sigma_{a}}$$

where $\mu_{d}$ , $\mu_{a}$ are the mean fixation duration and saccade amplitude, respectively, and $\sigma_{d}$ , $\sigma_{a}$ are the fixation duration and saccade amplitude standard deviation, respectively, computed over all *n* fixations to produce *n* (*K_i_*) coefficients. A *K* coefficient close to zero indicates relative similarity between fixation durations and saccade amplitudes. Whereas positive values of *K*_i_ show relatively long fixations followed by short saccade amplitudes, which indicate focal attention. In this case, attention is concentrated on a few areas of interest, specified by a central or peripheral cue. Conversely, negative values of *K*_i_ point towards relatively short fixations followed by relatively long saccades, suggesting ambient or diffuse attention (Unema et al., 2005). Here, visual attention is allocated to all regions of the visual field in near equal proportion (Heitz and Engle, 2007). While performing tasks novices typically demonstrate more focal attention, while experts distribute attention more evenly across the visual field. Task difficulty can prompt both groups to shift from focal to ambient viewing as demands increase (Lounis et al., 2021). Using GTE and K coefficients as visual attention metrics during FS training can allow the assessment of how instructional modalities influence attention and, ultimately, learning outcomes.

### 2.5 Motivation, immersion and affect

Immersion has been defined as “a state of deep mental involvement in which the individual may experience disassociation from the awareness of the physical world due to a shift in their attentional state” (Agrawal et al., 2020). Immersion is based on the extent to which visual displays support an illusion of reality that is inclusive (denoting the extent to which physical reality is shut out), extensive (the range of sensory modalities accommodated), surrounding (the size of the field of view), and vivid (the display resolution, richness, and quality) (Slater and Wilbur, 1997). Designers have longed to create FS that provide the most training transfer (Myers et al., 2018). Positive training transfer happens when performance in the aircraft is better than if there was no simulator training provided, as opposed to negative training transfer that happens when performance in the aircraft is poorer than if there was no pre-training at all (Lintern, 1991). Among other factors (e.g., simulator fidelity, presence, operator buy-in), increased immersion has been shown to drive positive training transfer (Alexander et al., 2005). Additionally, previous studies have demonstrated that high immersion increased user motivation and subsequently engagement (Dalgarno and Lee, 2010; Liu et al., 2017; Bailenson et al., 2008; Dede, 2009). According to the CATL, immersion functions as a motivational affordance by increasing inclusiveness and vividness, which leads to sustained engagement, which in turn promote deeper processing and persistence during practice (Agrawal et al., 2020; Slater and Wilbur, 1997). In fact, positive affect has been shown to facilitate motivation, and learning (Isen, 2004; Wu and Holsapple, 2014). In applied training, this increased motivation offers could be a plausible mechanism behind positive transfer observed with more immersive or well-designed FS systems (Callender et al., 2009; Myers et al., 2018; Valverde, 1973; Noetel et al., 2022). Therefore, FS properties that increase this dynamic between immersion and motivation could lead to improved learning performance during training.

### 2.6 Hypothesis development

Previous research in multimedia learning has shown that presenting instructional material in more than one modality fosters deeper learning, leading to enhanced retention and transfer performance (Mayer and Moreno, 1998; Moreno and Mayer, 1999; Ginns, 2005). However, no study has specifically tested the modality principle within the context of flight simulation (FS) training.

Aircraft performance data, including deviations in heading, altitude, and speed relative to the flight plan, can serve as indicators of a pilot’s learning state. Additionally, cognitive state has been broadly defined as the status of human cognitive processes and resources, encompassing perception, attention, cognitive effort, engagement, working memory, arousal, stress, and fatigue (Dirican and Göktürk, 2011). An impaired cognitive state during learning may not immediately manifest as a significant change in performance outcomes. However, systematically assessing a learner’s cognitive state throughout and at the conclusion of training may allow researchers to identify the optimal sensory modality for delivering FS instructions to student pilots.

Based on these premises, we hypothesize that *bimodal* (audio and text)*, unimodal-audio, and unimodal-text flight instructions will influence pilots’ cognitive learning states at different levels*. According to CTML and CATL, instructional modalities affect cognitive processes across three primary dimensions: cognitive load, visual attention, and motivation. These dimensions form the basis of the following three sub-hypotheses:

- Cognitive Load: *Bimodal (audio and text), unimodal-audio, and unimodal-text flight instructions will result in different levels of cognitive load, as reflected by subjective ratings and physiological indicators*.
- Visual Attention: *Instruction modality will influence pilots’ perceptual and attentional strategies, as measured by differences in gaze transition entropy (GTE) and focal-ambient attention dispersion*.
- Motivation and Affect: *Instruction modality will impact motivation and emotional engagement, evidenced by* var*iations in emotional valence, subjective motivation, and immersion*.

Prior research on data link communication suggests that different modalities influence learning performance differently. Specifically, textual and bimodal instructions have been associated with increased accuracy in executing navigational instructions (Helleberg and Wickens, 2003), while auditory instructions have demonstrated advantages in tasks where response time is a critical factor (Lancaster and Casali, 2008). Based on these findings, we expect similar learning mechanisms to be at play in this study.

Thus, we hypothesize that *instruction modality (bimodal, unimodal-audio, and unimodal-text) will generate differences in pilot learning performance, with the optimal modality fostering deeper learning and improved execution of flight objectives*.

## 3 Methods

This study used a between-subject experimental design to investigate the effects of sensory modality on pilots’ cognitive learning states and performance. In total thirty pilot students participated in the study, completing tasks in a simulated flight environment using Microsoft Flight Simulator 2020 (MFS 2020). Experimental conditions included *bimodal*, *unimodal-text* and *unimodal-audio* instructional modalities. A series of psychometric, performance, and physiological measures were used to assess learning in the flight tasks, perceived and experienced cognitive load, and perceived motivation. This section provides details on the ethical considerations, participants, experimental setup, apparatus, and statistical analyses employed.

### 3.1 Participants

Thirty pilot students were recruited at a pilot training and flight school in Quebec, Canada, to participate in this study, yielding a convenience (non-probability) sample. The final sample therefore comprised the 30 individuals who both satisfied the study’s inclusion/exclusion criteria and opted in. Convenience panels are frequently used in exploratory research and are judged acceptable at this stage, but the absence of random selection inevitably limits the generalizability of the results (Bornstein et al., 2013; Button et al., 2013). A sample of this size is typical of exploratory laboratory experiments that employ psychophysiological measures (Lamontagne et al., 2020). The inclusion criteria were: participants must be older than 18 years old and understand advanced spoken and written French or English, with some experience of aircraft flight. Participants were excluded if they had laser vision correction or astigmatism, a neurological or psychiatric diagnosis, or suffered from epilepsy. In a recruitment questionnaire, candidates indicated their previous use of various flight simulators, Total Flight Hours (TFH), and flight qualification (aircraft type). Participants were assigned as to control the level of expertise of each sensory modality experimental group. Novices (NOV) had TFH ranging from 25 to 100; intermediates’ (INT) TFH ranged from 101 to 200; and advanced’ (AD) TFH was over 200. Participants were separated into three groups. Table 1 shows the distribution and demographics among the three groups. All subjects had prior theoretical knowledge, and a good comprehension of the various information displayed on a standard cockpit instrument panel; had flight knowledge or experience related to manual interactions with the aircraft; had already flown a Cessna-152. Finally, all subjects had used a flight simulator but were first-time users of the Visual Flight Rules (VFR) module of MFS 2020 (Xbox Game Studios, 2020).

**Table 1:** Participant distribution and demographics.

Group	Total participants	NOV	INT	AD	Gender	Mean age (years)
Bimodal	11	4	5	2	11 M	24.6 ± 3.6
Unimodal – text	10	3	5	2	9 M, 1 F	24.1 ± 3.7
Unimodal – audio	9	3	4	2	7 M, 2 F	24.3 ± 6.0

### 3.2 Experimental design and procedure

This study used a between-subject experimental design to investigate the effects of sensory modality on flight instruction during an instructional flight task. Participants were assigned to one of three experimental conditions: (1) bimodal condition, which utilized the default settings of Microsoft Flight Simulator (MFS 2020), combining a synthesized speech virtual instructor and a textual flight objectives display; (2) unimodal-audio condition, which included only a synthesized speech virtual instructor without additional on-screen flight objectives; and (3) unimodal-text condition, which provided textual virtual instructor guidelines and flight objectives displayed on-screen.

**Figure 2:**
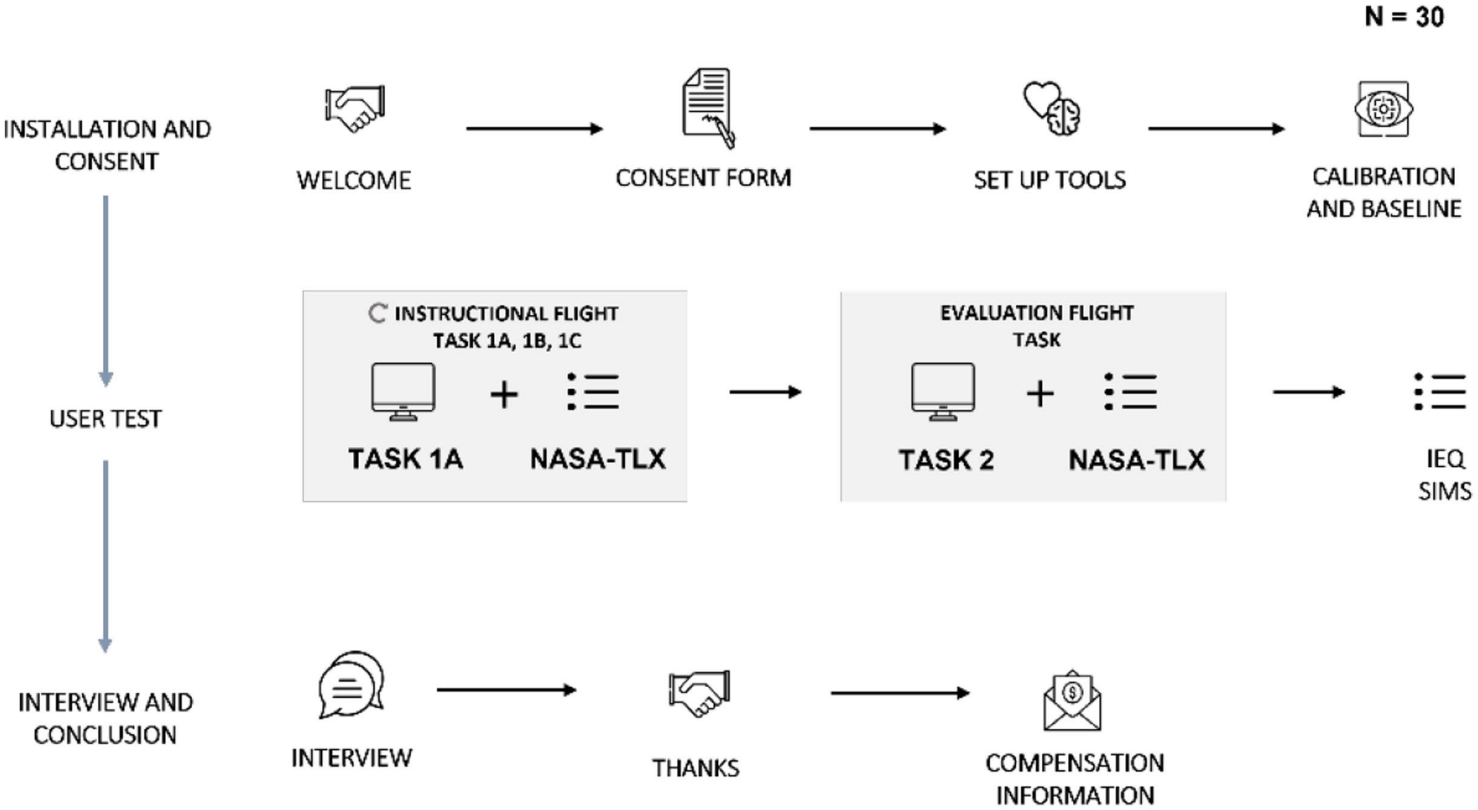
Experimental procedure.

A 90-min experiment, summarized in Figure 2, was conducted in a laboratory setting. After completing a consent form and undergoing a 7-point eye-tracking calibration, participants reviewed a task description (flight plan) on a computer screen. The simulation screen displayed a navigation log containing checkpoints, route times, and headings to navigate between Airports A and B. Following each flight task segment, participants completed the NASA-TLX subjective cognitive load questionnaire, while additional psychometric questionnaires (Immersion, IEQ; Motivation, SIMS) were completed after the experimental tasks. The experiment concluded with an interview to gather qualitative data on participants’ perceptions of their learning experience and the system’s strengths and weaknesses. Participants were then compensated $30 and were entered into a draw to win a prize valued at $600 (Microsoft Flight Simulator 2020 and an Xbox Series S).

### 3.3 Apparatus

The MFS 2020 software was used for this experiment. The simulation was presented on a 27-inch computer screen. The subjects controlled the aircraft with a yoke, a sidestick, two thrust levers, and a rudder. They could use a joystick on the yoke to change view and gain better visibility OTW in the VE. The participants’ screen was recorded, and their flight performance was assessed by an experienced pilot *post hoc* using the session recordings. The aircraft flown was a Cessna-152, which was depicted accordingly in high definition in the simulation. Eye movements were recorded at a sampling frequency of 60 Hz using the Tobii Pro Nano (Tobii, Stockholm, Sweden) eye tracker, which uses near-infrared diodes to identify the position of each eyeball in the three-dimensional space and to calculate the gaze point on the screen (Tobii Pro, 2021).

**Figure 3:**
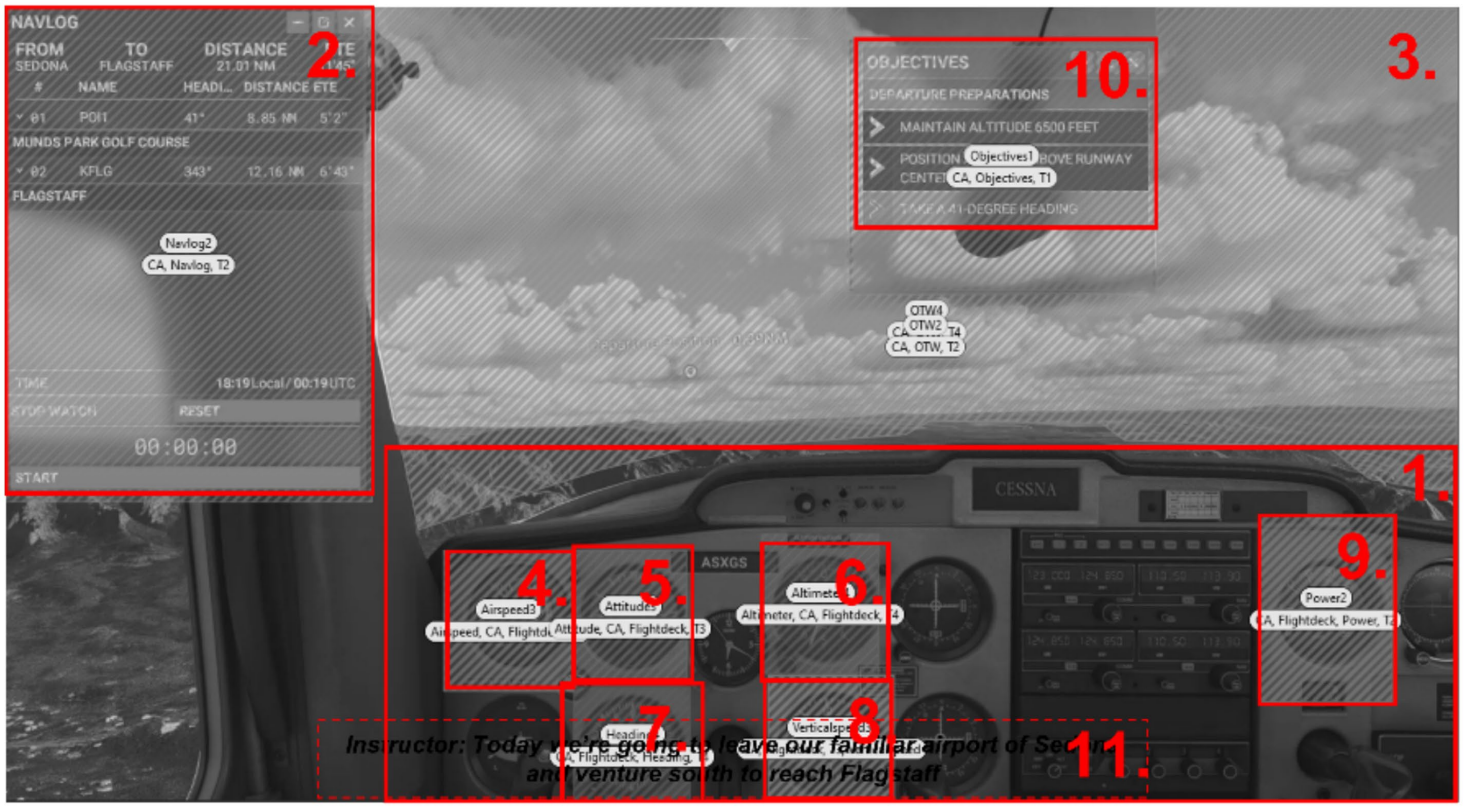
Overview of the ten different AOIs: 1. Flight deck (including AOIs 4–9), 2. Navigation Log, 3. Out The Window view, 4. Airspeed Indicator, 5. Attitude Indicator, 6. Altimeter Indicator, 7. Heading Indicator, 8. Vertical Speed Indicator, 9. Power Indicator, 10. Flight Objectives Display.

The cockpit was split into 8 to 10 AOIs corresponding to the flight instruments and instruction displays necessary for successful task completion, as shown in Figure 3. AOIs included a flight deck, a navigation log, and an external view (i.e., OTW). Two condition-specific AOIs were also analyzed: a flight objectives display (*bimodal*, *unimodal-text*) and a textual flight instructor (*unimodal-text*).

### 3.4 Simulated scenarios

Following a series of tests carried out with a flight instructor from the Cargair Ltée flight school, the VFR module developed by Asobo Studio was selected for this study (Xbox Game Studios, 2020). The training module presents moderate task difficulty, moderate task length, a familiar aircraft type, and various instruction modalities; flying in VFR requires pilots to allocate a portion of their visual–spatial attention outside the aircraft to locate landmarks, thus creating competition for attention when presenting other instructional material. The experimentation was separated into two main tasks. In the first “instructional flight task,” a participant flew from Sedona Airport to Flagstaff-Pulliam Airport with the help of their virtual instructor. In the second “evaluation flight task,” a participant flew from the Flagstaff-Pulliam Airport back to Sedona Airport during a solo flight without a virtual instructor. During this second flight evaluation task, no instructions were provided to pilots. Thus, the task was identical across experimental conditions. Subjects were informed that the first task’s flight objectives would be evaluated during the evaluation flight task. Hence, the second task aimed at assessing how the modality of flight instructions during an instructional flight task led to training outcomes during an evaluation flight task. The description of the flight scenario is presented in Table 2.

**Table 2:** Tasks descriptions.

Flight task	Flight task segment	Description
Instructional flight task From Airport A (Sedona) to Airport B (Flagstaff-Pulliam 30 min)	Departure	Via instructions presented throughout the task, a participant learns how to take off from Airport A, how to climb to a prescribed altitude, and how to maintain a prescribed heading.
	Navigation	Via instructions presented throughout the task, a participant learns how to find a *checkpoint* during a flight, and how to start the stopwatch to calculate the duration of a flight segment to compare it with the *Navigation Log*.
	Arrival	Via instructions presented throughout the task, a participant learns how to *integrate a circuit* when arriving at Airport B, and how to land on a specified *Runway*.
Evaluation flight task From Airport B (Flagstaff-Pulliam) to Airport A (Sedona) 30 min	N/A	In a first solo flight, a participant is required to take off from Airport B, to climb to a cruising altitude and heading; to use diverse methods of navigation (*Landmark Navigation* and *Dead Reckoning*) to navigate to Airport A; to arrive at Airport A, integrate its circuit and land.

The instructions provided to users throughout the session took two forms. First, a synthesized or textual speech virtual instructor informally provided instructions to pilots. The synthesized speech instructor could be heard through the computer speakers, whereas the textual speech instructor could be read directly on-screen. The virtual instructor was responsive to participant behaviors and would, therefore, repeat instructions, bring back a user to previous flight objectives, or explain participant mistakes if needed. Second, a flight objective display appeared in the upper-right corner of the UI simulator screen and would summarize concise flight objectives in real-time. Flight objectives that had to be met and maintained (e.g., “Maintain 8,000 ft.”) would dynamically appear/disappear on the screen signaled in green when correctly performed by participants, whereas flight objectives that had to be met but not maintained (e.g., “Reach 8,000 ft.”) would be successively displayed. All experimental tasks were performed linearly by participants to reproduce a real-world flight setting (i.e., departure to landing; Airport A to B and back). The learning performance was assessed at the task level (i.e., instructional flight task, evaluation flight task) and at the flight segment level (i.e., departure, navigation, arrival) for the instructional flight task, where each flight objective displayed in the simulator UI window was evaluated. Performance-dependent variables included speed, altitude, heading, power, navigation, and “pass or fail” flight objectives.

### 3.5 Measures

Four key constructs were used: cognitive load, visual attention, motivation, and learning performance. Each construct was assessed using multiple measures, which are summarized in Table 3.

**Table 3:** Summary of measures.

Construct	Measures	Operationalization
Learning performance	a. Subjective “overall performance” b. Observed in-flight performance	a. NASA-TLX (Hart and Staveland, 1988) b. Observed in-flight performance
Cognitive load	a. Perceived Cognitive load b. Experienced cognitive load	a. NASA-TLX (Hart and Staveland, 1988) b. PCPD from baseline (Attard-Johnson et al., 2019)
Visual attention	a. Visual attention dispersion b. Visual transition entropy	a. Focal-ambient K coefficients (Krejtz et al., 2016) b. GTE/Hmax (Shiferaw et al., 2019)
Affect and motivation	a. Experienced emotional valence b. Perceived motivation c. Perceived immersion	a. FaceReader (Loijens and Krips, 2018) b. SIMS (Guay et al., 2000) c. IEQ (Jennett et al., 2008)

#### 3.5.1 Learning performance

An experienced pilot watched the participant’s screen recordings using the Tobii Pro Lab video replay function to assess flight performance. Each flight objective was marked as « 0 » (i.e., failure), « 1 » (i.e., partial success) or « 2 » (i.e., success). When the FS made the user start over at a previous flight objective, the unsuccessful objective was marked as failed. In this case, we kept the score of the first trial for each flight objective performed twice and started scoring normally when the participant was past the objective, which led to the backtracking of the simulation. For the “Maintain altitude/heading/speed” objective types, « 0 » was assigned if the flight objective in the simulator window appeared green less than 25% of the time, « 1 » was assigned if it appeared green 25–75% of the time, and « 2 » if it appeared green more than 75% of the time. If a participant was not able to finish a task, each flight objective not performed was marked as a failure. Weights were applied to flight objectives to fit the score computed by MFS 2020. During the instructional flight task, each flight segment (i.e., departure, navigation, and landing) gave a total score of 20 Pts. The instructional flight task and the evaluation flight task gave total scores of 60 Pts.

#### 3.5.2 Cognitive load

Cognitive load was assessed using both perceived and experienced measures. The National Aeronautics and Space Administration-Task Load Index (NASA-TLX) was used to assess perceived cognitive load. The NASA-TLX is a well-known and often-used multi-dimensional rating scale (Hart and Staveland, 1988) to measure cognitive load through six items: mental demand, physical demand, temporal demand, overall performance, effort, and frustration. The experienced cognitive load of pilots was measured using PCPD, which is measured by calculating the difference between the pupil diameter measured during a task and a pre-stimulus baseline level, divided by the pre-stimulus baseline level. This baseline typically corresponds to an average value over a few seconds period of pupil diameter data measured before the experiment (Attard-Johnson et al., 2019). In this experiment, the last 10 s of each task were used as a baseline to ensure that the screen lighting condition was that of the simulator and to synchronize with simulator view switching after the last task flight objective was completed by a participant.

#### 3.5.3 Visual attention

Visual attention was evaluated through eye-tracking measures of visual transition entropy and visual attention dispersion. The eye-tracking data was pre-processed in Tobii Pro Lab v.161 (Tobii, Stockholm, Sweden). As participants could switch views in the aircraft, AOIs were coded manually after data collection. Event markers were positioned at the start and end of each experimental task for each participant. Task duration varied consequently to participant actions. The AOIs data were extracted from the raw data, and the Tobii Pro Lab Tobii I-VT fixation filter was used, which is based on the work of Salvucci and Goldberg (2000) and Komogortsev et al. (2010). Fixations inferior to 60 ms were discarded, and a velocity threshold of 30 degrees/s was used. To compute the GTE and K coefficients, home-built scripts were coded following the methodology described in Shiferaw et al. (2019) and Krejtz et al. (2016), respectively. From this, GTE/H_max_ and focal-ambient K coefficient were assessed using the method described in 2.2.2.

#### 3.5.4 Affect and motivation

To assess the motivational and emotional states and of pilots, affect, subjective motivation and subjective immersion were used. Affect was measured through emotional valence, which was detected using the facial video stream of each participant recorded with a webcam which was analyzed in real-time using FaceReader v6.0 (Noldus Information Technology, 2015), using facial emotion recognition. FaceReader analyzes participants’ facial movements to detect six emotions. It then calculates emotional valence as the intensity of positive emotion minus the intensity of negative emotions, which renders a score between 0 (negative) to 1 (positive) (Loijens and Krips, 2018; Ekman and Friesen, 1978). Subjective motivation was measured using the Situation Motivational Scale (SIMS), a 16-item scale developed by Guay et al. (2000) that includes constructs of intrinsic motivation, identified regulation, external regulation, and motivation. Pilots’ perceived immersion was measured using the Immersive Experience Questionnaire (IEQ), a 31-item scale developed by Jennett et al. (2008) that includes affective, cognitive, real-world dissociation, challenge, and control components while playing a game. These psychometric questionnaires evaluating subjective immersion and motivation were collected only once following the evaluation flight task to minimize the negative effects of a lengthy experimental session (e.g., boredom, fatigue) and to prevent participants’ responses from being affected by the redundancy of questions.

### 3.6 Statistical analysis

All data were analyzed using the statistical software SAS 9.4 (SAS Institute Inc., 2013) with custom homebuilt scripts. The synchronization of the apparatus and event markers was achieved by the Observer XT software, which allowed the triangulation of user data with Cube HX (Léger et al., 2019). All analyses were either performed at the flight task level (i.e., “learning” and “evaluation”) or at the flight segment level (i.e.,“departure,” “navigation” and “arrival” segments) of the instructional flight task. The statistical tests are based on data aggregated (i.e., one data point) per participant and task for all analyses performed at the flight task and flight segment levels. The IEQ and SIMS were assessed once after the instructional flight task and tested using a linear regression with random intercept model. *p*-values were adjusted for multiple comparisons using the Holm-Bonferroni method. A Kruskal Wallis Test was used to evaluate if the performance differed by condition at the flight task and flight segment levels. A repeated measures ANOVA was performed for each of the following dependent variables to assess the effects of the sensory modalities at the flight task and flight segment levels (Holm-Bonferroni corrected): PCPD from participant baseline, emotional valence, GTE/H_max_, and focal-ambient K coefficient. A Kruskal-Wallis Test was used to assess the NASA-TLX results at the flight task level, and a linear regression with random intercept model (Holm-Bonferroni corrected) was performed to assess if the DV differed by condition at the flight segment level. In line with standard practice in psychology and HCI research, we set a significance threshold of *α* = 0.05 for detecting statistically significant effects. However, findings with *p*-values between 0.05 and 0.10 were reported as a trend. This approach in also commonly used in psychology and HCI, especially in exploratory research (Cairns, 2019; Olsson-Collentine et al., 2019).

## 4 Results

Results are reported in this section. First, the pilot learning performance was compared between modalities. Then, modalities were compared by the cognitive states, specifically cognitive load, visual attention, motivation and immersion. In each case, results at the *task level* are first reported to assess the effect of the modalities during an instructional flight and an evaluation flight task. Second, at the *flight segment level* to evaluate the effects of the modalities during the departure, navigation, and landing parts of the instructional flight task.

### 4.1 Learning performance

**Figure 4:**
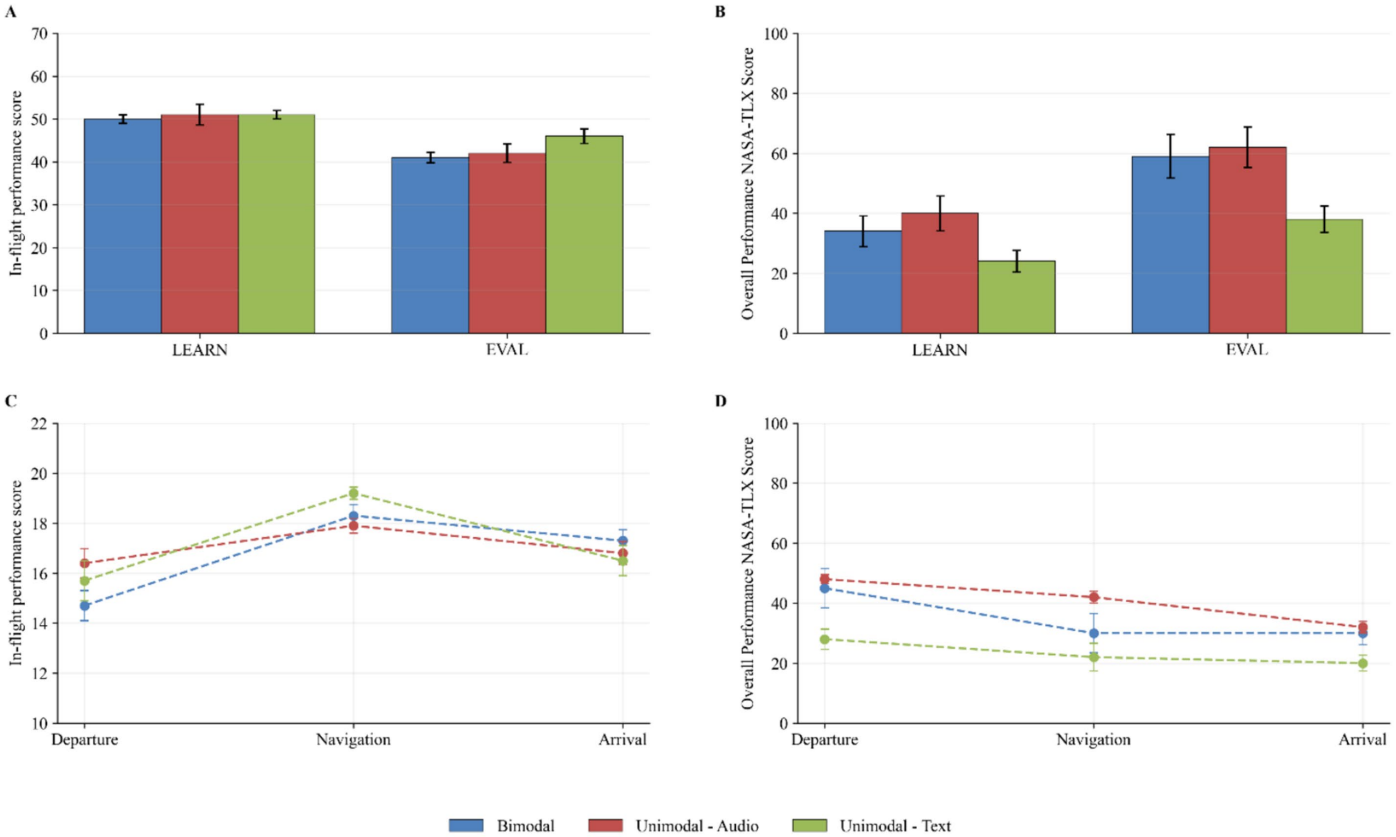
Mean in-flight performance scores and overall performance NASA-TLX scores for each modality group; Bimodal (blue), Unimodal-Audio (red/orange), and Unimodal-Text (green). In **(A)**, the in-flight performance scores for the LEARN and EVAL tasks are shown. In **(B)**, the corresponding NASA-TLX scores are shown. In **(C)**, the in-flight performance scores by flight segment are shown and in **(D)**, the the NASA-TLX scores across the same segments are shown. In-flight performance scores reflect observational ratings, with higher values indicating better performance. NASA-TLX scores reflect subjective workload ratings of performance, with higher values indicating poorer perceived performance. Error bars represent ±1 standard error of the mean (SE).

Mean objective learning performance ratings (and standard deviations) for instructional flight task and evaluation flight task in Figure 4A. There was no significant difference between modalities in performance in both the instructional flight task (*X*^2^ = 0,512, df = 2, *p* = 0,774, *ε*^2^ = 0.001) and the evaluation flight task (*X*^2^ = 1,1,455, df = 2, *p* = 0,564, *ε*^2^ = 0.002). Mean perceived learning performance (retrieved from the NASA-TLX) is shown in Figure 4B. A trend was detected in flight instruction modality on pilots’ perceived overall performance for the instructional flight task (*F*(2, 52) = 2.5, *p* = 0.0917, *η*^2^ = 0.088) but not the evaluation flight task (*F*(2, 23) = 2.27, *p* = 0.1258, *η*^2^ = 0.165), albeit with a strong effect size. In both cases, the difference was notable as *bimodal* and *unidomal-audio* modalities showed higher perceived performance than *unimodal-text*. The mean objective performance (and standard deviation) at the flight segment level are shown in Figure 4C, and the subjective performances in Figure 4D. Results did not reveal a significant difference in objective performance between the three modality groups during the “Departure” flight segment (*X*^2^ = 1.143, df = 2, *p* = 0.565, *ε*^2^ = 0.002), the “Navigation” flight segment (*X*^2^ = 0.253, df = 2, *p* = 0.282, *ε*^2^ = 0.001), and the “Arrival” flight segment (*X*^2^ = 0.2499, df = 2, *p* = 0.883, *ε*^2^ = 0.001) of the instructional flight task. Similarly, a Kruskal Wallis Test did not reveal any statistically significant difference between the three conditions for the “Departure” flight segment (*X*^2^ = 2.627, df = 2, *p* = 0.269, *ε*^2^ = 0.023), the “Navigation” flight segment (*X*^2^ = 3.363, df = 2, *p* = 0.186, *ε*^2^ = 0.050), and the “Arrival” flight segment (*X*^2^ = 2.922, df = 2, *p* = 0.232, *ε*^2^ = 0.034) of the instructional flight task.

### 4.2 Cognitive load

**Figure 5:**
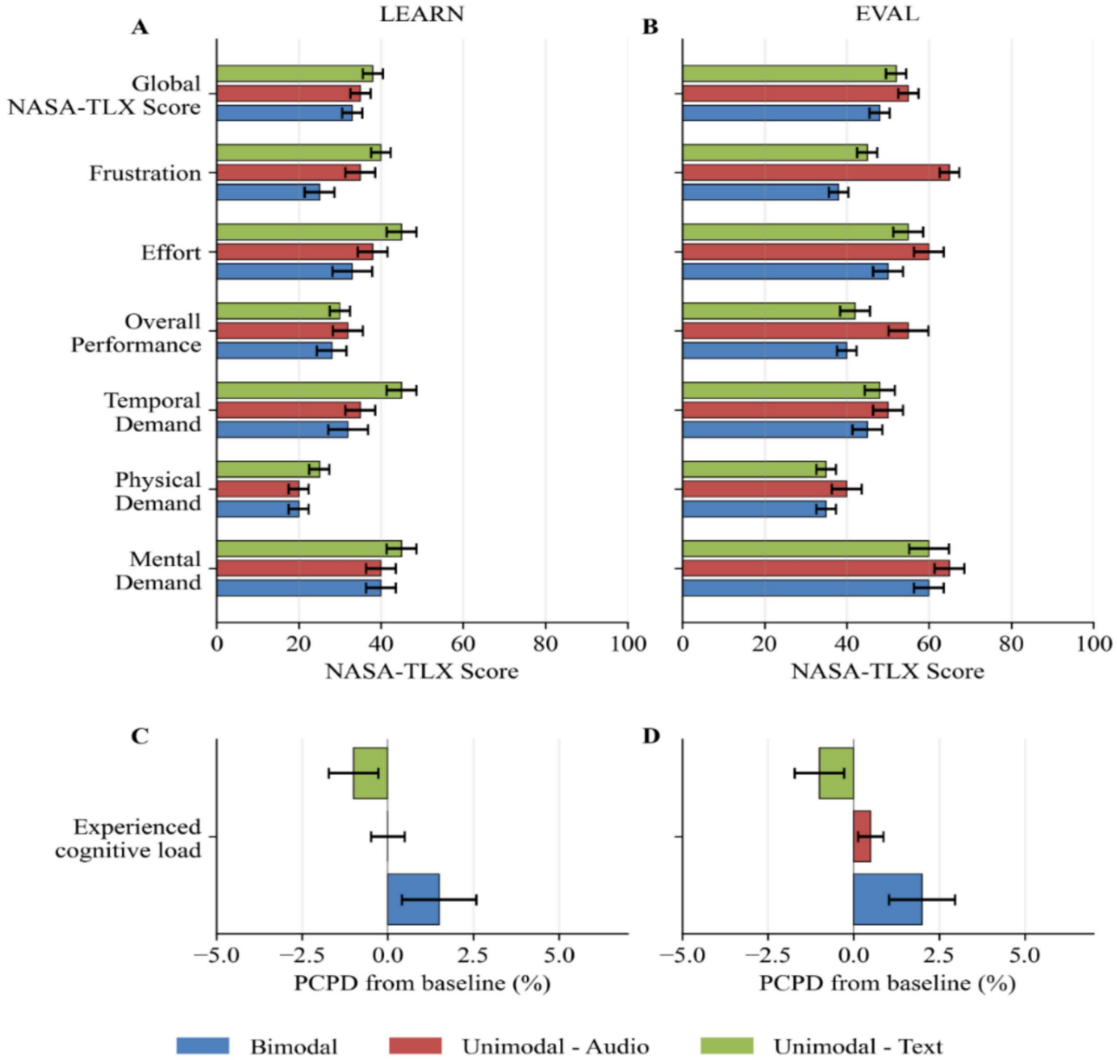
Mean in-flight performance scores and overall performance NASA-TLX scores for each modality group; Bimodal (blue), Unimodal-Audio (red/orange), and Unimodal-Text (green). In **(A)**, the in-flight performance scores for the LEARN and EVAL tasks are shown. In **(B)**, the corresponding NASA-TLX scores are shown. In **(C)**, the in-flight performance scores by flight segment are shown and in **(D)**, the the NASA-TLX scores across the same segments are shown. In-flight performance scores reflect observational ratings, with higher values indicating better performance. NASA-TLX scores reflect subjective workload ratings of performance, with higher values indicating poorer perceived performance. Error bars represent ±1 standard error of the mean (SE).

Average perceived cognitive load scores for each modality for the instructional flight task are shown in Figure 5A and the evaluation flight task in Figure 5B. A two-tailed Kruskal-Wallis Test revealed that the NASA-TLX global score, nor the NASA-TLX individual items results (i.e., mental demand, physical demand, temporal demand, overall performance, effort and frustration) significantly differed across sensory modality conditions during the instructional flight task and during an evaluation flight task. The average experienced cognitive load is shown in Figure 5C. A type III ANOVA revealed no significant effect of modality on experienced cognitive load during either the instructional flight task (*F*(2, 27) = 0.89, *p* = 0.4208, *η*^2^ = 0.062) or the evaluation flight task (*F*(2, 24) = 1.65, *p* = 0.2126, *η*^2^ = 0.121). However, descriptively, the unimodal-text condition was associated with lower cognitive load in both tasks, as indicated by more negative values. Regarding the individual flight segments, there were no significant differences in experienced cognitive load between modality groups during departure, navigation, or arrival, as shown by both a one-way ANOVA (Departure: *F*(2, 24) = 1.73, *p* = 0.199, *η*^2^ = 0.126; Navigation: *F*(2, 24) = 0.72, *p* = 0.498, *η*^2^ = 0.057; Arrival: *F*(2, 24) = 1.51, *p* = 0.240, *η*^2^ = 0.112) and a two-way ANOVA (Departure: *F*(2, 23) = 0.60, *p* = 0.5582, *η*^2^ = 0.051; Navigation: *F*(2, 23) = 0.15, *p* = 0.858, *η*^2^ = 0.013; Arrival: *F*(2, 23) = 0.21, *p* = 0.8159, *η*^2^ = 0.018).

### 4.3 Visual attention

**Figure 6:**
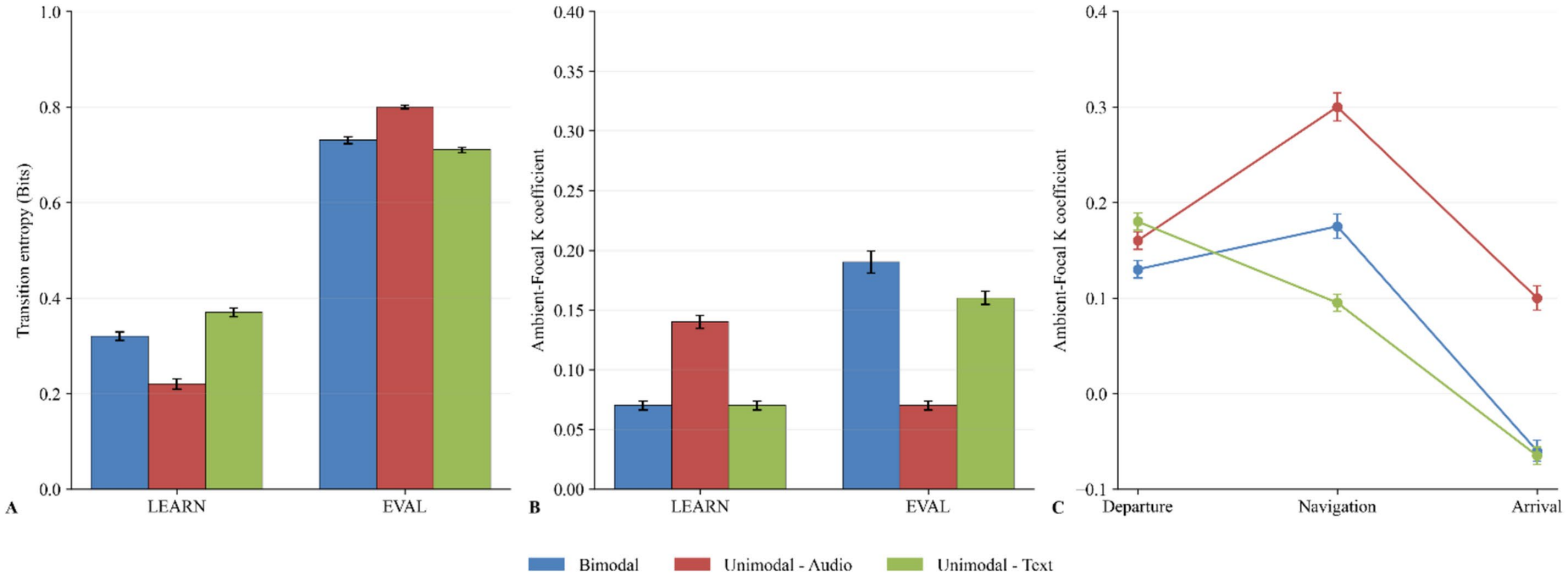
Behavioral and cognitive measures across learning and evaluation phases for bimodal (blue), unimodal-audio (orange), and unimodal-text (green) conditions. **(A)** Transition entropy (GTE/Hmax) scores during LEARN and EVAL phases for each modality condition (bimodal, unimodal-audio, unimodal-text). Higher values indicate more exploratory behavior, while lower values suggest more deterministic scanning patterns. **(B)** Ambient-Focal coefficient K comparing the relative distribution of ambient and focal attention allocation between LEARN and EVAL phases. **(C)** Evolution of the Ambient-Focal coefficient K across flight segments (Departure, Navigation, Arrival). Error bars represent standard error of the mean (SEM) in all panels.

Visual transition entropy (GTE/Hmax) results are presented in Figure 6A. A two-way type III ANOVA did not reveal that there was a statistically significant difference in visual transition entropy between at least two sensory modality conditions during the instructional flight task (*F*(2, 24) = 0.23, *p* = 0.798, *η*^2^ = 0.019). However, results revealed a statistically significant difference between at least two sensory modality conditions during the evaluation flight task (*F*(2, 21) = 12.07, *p* = 0.0003, *η*^2^ = 0.535). Pairwise comparisons indicate that the mean value of GTE/h_max_ was significantly different between the bimodal condition and the unimodal-audio condition (*F*(1, 15) = 13.01, *p* = 0.005, *η*^2^ = 0.464) and between the audio condition and the text condition (*F*(1, 14) = 33.39, *p* < 0.0001, *η*^2^ = 0.705). A two-way type III ANOVA did not reveal that there was a statistically significant difference in visual transition entropy between at least two sensory modality conditions during the departure flight segment (*F*(2, 24) = 0.65, *p* = 0.533, *η*^2^ = 0.051), the navigation flight segment (*F*(2, 21) = 0.52, *p* = 0.605, *η*^2^ = 0.047) and the arrival flight segment (*F*(2, 21) = 0.89, *p* = 0.426, *η*^2^ = 0.078) of the instructional flight task.

Focal-Ambient K coefficients are shown in Figure 6B. The mean ratings show that coefficients across tasks and conditions were above zero. Analysis showed that there was no statistically significant difference in K coefficients between the three modalities during the instructional task (*F*(2, 29) = 2.06, *p* = 0.145, *η*^2^ = 0.124). Still, the K coefficient was notably higher in the *audio unimodal* modality. A trend was found in the evaluation task (*F*(2, 26) = 2.92, *p* = 0.072, *η*^2^ = 0.138), as K coefficients in the bimodal and unimodal-text group were higher than the audio condition. The K coefficients at the segment level are shown in Figure 6C. There were no statistically significant differences in K coefficients between the three experimental conditions for the departure (*F*(2, 26) = 0.66, *p* = 0.5268, *η*^2^ = 0.048) segments while a trend was observed in the arrival (*F*(2, 26) = 2.9, *p* = 0.073, *η*^2^ = 0.182) segment. A two-way type III ANOVA revealed a main effect of the flight instruction modality on the arrival flight segment K coefficient results (*F*(2, 26) = 3.61, *p* = 0.0413, *η*^2^ = 0.217). However, the pairwise comparisons did not reveal any statistically significant differences between the bimodal and unimodal-audio conditions (*F*(1, 19) = 3.41, *p* = 0.161, *η*^2^ = 0.217) and the bimodal and unimodal-text conditions (*F*(1, 17) = 1.24, *p* = 0.281, *η*^2^ = 0.152), but a trend was observed in the unimodal-audio and unimodal-text conditions (*F*(1, 16) = 5.76, *p* = 0.0867, *η*^2^ = 0.265).

### 4.4 Motivational states

**Figure 7:**
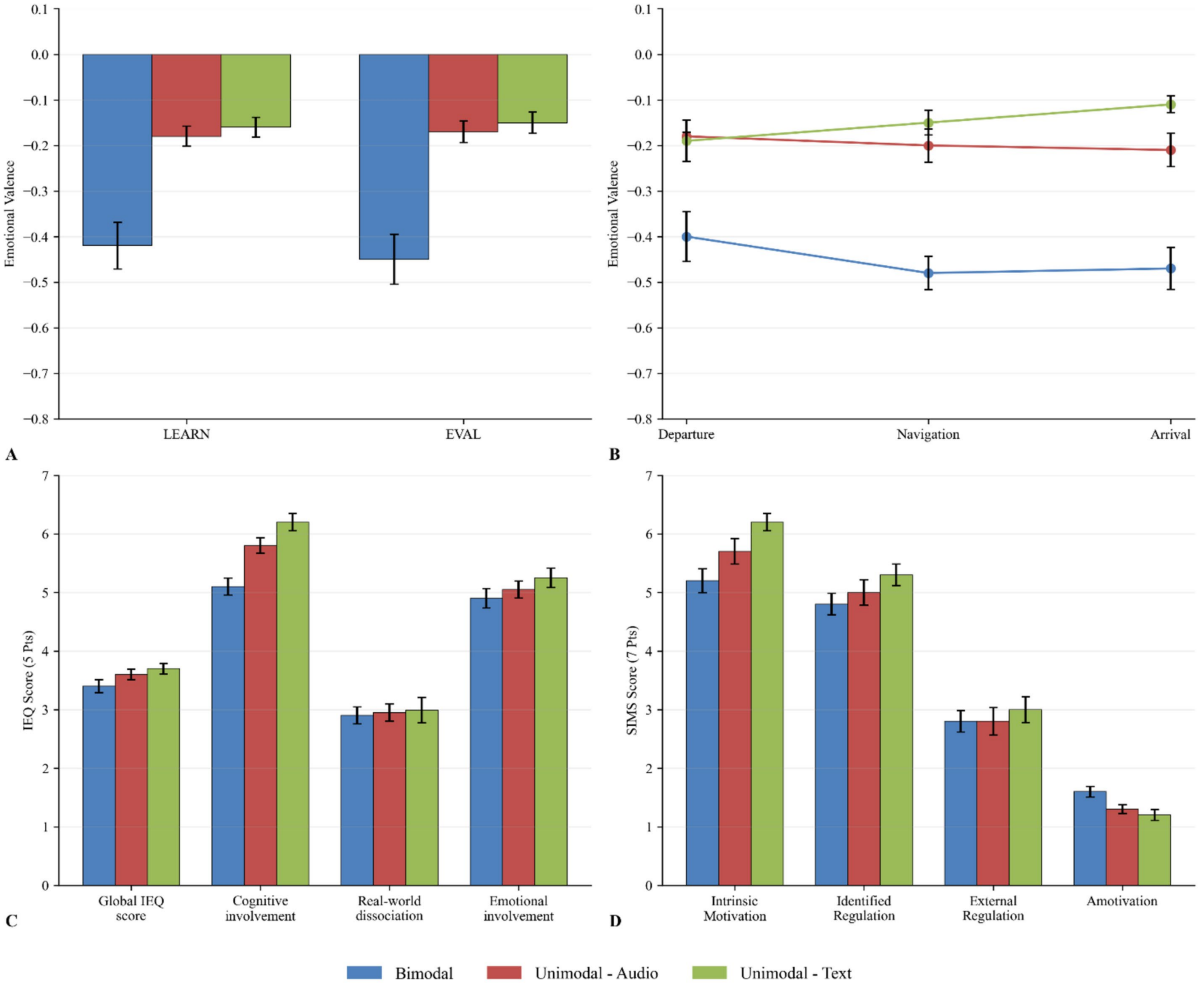
Emotional responses, immersion, and motivation across modality conditions. **(A)** Mean emotional valence per modality group during the LEARN and EVAL phases. More negative values indicate more negative emotional responses during the flight tasks. **(B)** Evolution of emotional valence across flight segments (Departure, Navigation, Arrival) for each modality group, showing how emotional responses develop throughout the flight path. **(C)** Immersion scores measured by the IEQ (Immersive Experience Questionnaire) across four components for each modality group. Higher scores indicate stronger immersive experiences on a 5-point scale. **(D)** Motivation scores measured by the SIMS (Situational Motivation Scale) across four regulatory components for each modality group on a 7-point scale. Higher scores on intrinsic motivation and identified regulation indicate more self-determined types of motivation, while higher scores on external regulation and amotivation indicate less self-determined types. Error bars represent standard error of the mean (SEM) in all panels.

Average emotional valence scores are shown in Figure 7A by modality for both instruction and evaluation tasks. All mean emotional valence scores were negative across all conditions and tasks. Results revealed a significant main effect of the modality on the emotional valence for the instructional flight task (*F*(2, 25) = 4.89, *p* = 0.016, *η*^2^ = 0.218). Pairwise comparisons showed trends that the text-only (*F*(1, 17) = 6.01, *p* = 0.0759, *η*^2^ = 0.161) and audio-only (*F*(1, 17) = 4.99, *p* = 0.078, *η*^2^ = 0.227) were higher than the bimodal group. No difference was found between both unimodal conditions (*F*(1, 16) = 0.15, *p* = 0.701, *η*^2^ = 0.009). A type III ANOVA revealed the main effect of the modality on emotional valence for the evaluation flight task (*F*(2, 22) = 5.71, *p* = 0.010, *η*^2^ = 0.342). In this case, the pairwise comparisons type III ANOVAs indicated that the emotional valence was significantly higher for the text condition when compared with the bimodal condition (*F*(1, 15) = 7.33, *p* = 0.049, *η*^2^ = 0.329), while a trend was observed where unimodal-audio was higher than bimodal (*F*(1, 15) = 5.04, *p* = 0.080, *η*^2^ = 0.251). Once again, no difference was observed between the unimodal conditions (*F*(1, 14) = 0.66, *p* = 0.429, *η*^2^ = 0.045). Emotional valence within flight segments scores are shown in Figure 7B. A main effect of sensory modality on emotional valence was found for each of the three flight segments of the instructional flight task: the departure segment (*F*(2, 22) = 3.79, *p* = 0.039, *η*^2^ = 0.256), the navigation segment (*F*(2, 22) = 5.74, *p* = 0.01, *η*^2^ = 0.343), and the arrival segment (*F*(2, 22) = 6.04, *p* = 0.008, *η*^2^ = 0.354). Pairwise comparisons revealed significant differences in mean emotional valence between the *bimodal* and the *unimodal-text* condition during the navigation flight segment (*F*(1, 15) = 7.49, *p* = 0.0479, *η*^2^ = 0.333) and the arrival flight segment (*F*(1, 15) = 8.65, *p* = 0.03, *η*^2^ = 0.336).

Average perceived immersion scores are shown in Figure 7C. No significant differences were found between conditions (*F*(2, 23) = 0.91, *p* = 0.415, *η*^2^ = 0.073), including its sub-scale factors cognitive involvement (*F*(2, 23) = 0.96, *p* = 0.3981, *η*^2^ = 0.077), real-world dissociation (*F*(2, 23) = 0.24, *p* = 0.7886, *η*^2^ = 0.020) and emotional involvement (*F*(2, 23) = 0.76, *p* = 0.479, *η*^2^ = 0.062). However not significant, a trend across the scale’s sub-factors points in favor of higher perceived immersion for the *unimodal-text* condition. The sub-scale factors of challenge and control were not used, as Cronbach’s alpha did not reach higher than 0.1 and 0.6, respectively. Internal consistency for the IEQ (Cronbach’s *α*): Global IEQ score (0.87), Cognitive involvement (0.78), Real-world dissociation (0.74), Emotional involvement (0.72); and for the SIMS: Intrinsic motivation (0.88), Identified regulation (0.70), External regulation (0.67), Amotivation (0.89). The sub-scale factor of real-world dissociation had a Cronbach’s alpha of 0.4 with its seven original items; only three items were therefore considered (i.e., *To what extent did you forget about your everyday concerns? To what extent did you feel as though you were separated from your real-world environment? To what extent was your sense of being in the game environment stronger than your sense of being in the real world?*).

Average perceived motivation scores are shown in Figure 7D. There were no main effect found of the modality across groups on the perceived motivation of pilots for the scale’s factors of intrinsic motivation (*F*(2, 23) = 2.13, *p* = 0.142, *η*^2^ = 0.156), identified regulation (*F*(2, 23) = 0.6, *p* = 0.557, *η*^2^ = 0.050), external regulation (*F*(2, 23) = 0.03, *p* = 0.974, *η*^2^ = 0.003) and amotivation (*F*(2, 23) = 1.01, *p* = 0.381, *η*^2^ = 0.081). A consistent trend points towards higher perceived motivation for the text unimodal condition. All factors showed acceptable Cronbach’s alphas. However, the factor of external regulation reported Cronbach’s alpha of 0.62 with its four original items; therefore, only three items were considered (i.e., *Because I am supposed to do it*.; *Because it is something that I have to do*.; *Because I do not have any choice.*).

## 5 Discussion

Flight simulator training has become indispensable in aviation as controlled environments where pilots can learn complex tasks without incurring real-world risks (Myers et al., 2018). As automated FS instructions are increasingly used, questions arise related to how best to design these virtual teaching systems and their impacts on pilot learning performance and their cognitive and emotional states. In this study, thirty student-pilots completed a guided instructional flight followed by an unguided evaluation flight within a flight simulator. Three instructional modalities were compared (unimodal-text, unimodal-audio, and bimodal with audio and text) to assess their impact on flight-school students’ learning performance, cognitive load, visual attention, and motivational states using self-reported, psychophysiological and performance-based metrics. Overall, no statistical differences were found in flight performance across modalities. While pilots’ self-ratings favored the bimodal and audio-only formats over text-only, affect was higher in the text-only condition. Visual scanning was more efficient in the text and bimodal conditions. Experienced and self-reported cognitive load were comparable among groups,

The similar objective and subjective performance in all three modalities support that each promotes behavioral competence equally well. However, this may be due to the relatively short tasks, as both training and evaluation were 30 min each. It may be that differences are observed over repeated or longer training tasks. For instance, there was a notable trend showing lower perceived performance in the text-only condition. This is in line with previous research showing that text can heighten error salience and thus depress self-evaluation (Lancaster and Casali, 2008). It may be that this increases or dissipates over time in longer tasks. Nonetheless, this difference between both measures further emphasizes the importance of using objective and subjective metrics when evaluating training outcomes. Similarly, both subjective and experienced cognitive load did not change significantly across modalities. Pupil size was descriptively lower for the text-based condition, which may be because textual instructions remain on-screen and pilots could pace themselves and avoid interruptions from audio information, reducing split-attention effects (Mayer, 2005). Still, this observation was purely descriptive, and it remains to be seen if this difference increases in longer tasks. Therefore, since the effects of modalities on learning performance and cognitive load could change over time and future research should consider longitudinal designs that track whether subtle modality advantages translate into greater retention in longer or repeated tasks and even transfer to real-world flight operations.

Furthermore, eye-tracking data showed that gaze-transition entropy was highest for audio-only trainees in the evaluation flight, indicating more random scanning, whereas text and bimodal groups adopted more deterministic patterns. This lower entropy is typically associated with schema-driven expertise and aligns with the focus on the textual information even if audio input was also presented (Diaz-Piedra et al., 2019; Lounis et al., 2021). A similar result is seen with ambient–focal K coefficients, which suggest that audio learners began with a focal strategy during instruction, likely because fewer on-screen cues allowed them to concentrate on instruments, then shifted toward ambient scanning in evaluation; the reverse trend appeared for text and bimodal pilots. Because shifting between cockpit instruments, out-the-window scans, and textual instructions is highly complex, future studies could explore more immersive setups (e.g., VR headsets or 360° displays) and measure in real-time, scanning strategies and gaze patterns in more intricate flight tasks. Additionally, controlling familiarity with the flight route may reduce extraneous visual search behavior.

Interestingly, the text-only condition showed higher emotional valence showed significantly more positive affect for text pilots during evaluation. Although SIMS motivation and IEQ immersion scores did not reach statistical significance, descriptive trends favoured the text-only modality for intrinsic motivation and immersion. This aligns with previous findings that reading offers a sense of autonomy and control, which can enhance emotional states (Moreno, 2006). However, these measures can be influenced by individual differences in reading speed or preference for auditory cues. Future research should manipulate different text complexities or use examine longer or more demanding simulator sessions. It may be that this increased affect for textual information may reduce over time, where audio or bimodal modalities will become preferred.

Overall, these results provide nuanced and detailed information of how instructional modality shapes pilots’ cognitive and affective processes. Although participants in all three conditions achieved similar objective performance levels, the text-only and bimodal modalities displayed more organized visual scanning while the audio-only modality showed more focal scanning. Benefits in emotional states were also observed with the text-only group. These findings align with evidence that textual instructions can reduce auditory pre-emption effects and facilitate stable reference points [20] but also point towards positive results for bimodal modality. Importantly, all participants, regardless of prior flight experience, were first-time users of Microsoft Flight Simulator 2020, a factor that may have amplified the initial positive affect toward text cues through a short-lived novelty effect (Tsay et al., 2020; Miguel-Alonso et al., 2024). Longitudinal evidence further suggests that the early affective boost from text may wane over successive sessions, after which trainees often prefer audio or bimodal presentations that sustain engagement without reading fatigue (Pattemore and Muñoz, 2024). Although the focus of this study was the effect of instructional modality, future studies could increase the sample of participants to test expertise-by-modality interactions and determine whether cognitive, emotional, and attentional responses diverge as pilots accrue experience. Accordingly, future research should track modality preferences over extended training blocks and probe whether expertise moderates these trajectories. Still, these results reveal an important schism between overt performance and covert cognitive processes: pilots flew equally well under all modalities, yet their attentional, load, and affective states diverged markedly. Such dissociations echo earlier warnings that behavior alone can mask latent overload or motivational decline (Charles and Nixon, 2019). Similarly, both the study therefore reinforces the necessity of a triangulated measurement strategy using objective behavioral indices, subjective self-reports, and physiological/eye-movement markers of cognitive and behavioral processes (Brunken et al., 2003; DeLeeuw and Mayer, 2008).

Importantly, these findings refine how CTML and CATL apply in high-element-interactivity flight tasks. While objective performance was comparable across modalities and text/bimodal showed lower transition entropy during evaluation (suggesting more efficient selection/organization), this pattern coexisted with minimal differences in perceived load. Notably, the expected bimodal advantage was not universal: text-only often matched or exceeded bimodal on attentional and affective markers, particularly in self-paced phases. This divergence from classical modality/redundancy predictions implies that, under time pressure and dense displays, added redundancy can introduce split-attention and transience costs that offset benefits (Mayer and Pilegard, 2005; Ginns, 2005). Therefore, it may be that effects are phase- and process-sensitive in which modality shapes attention, cognitive load, and affect/motivation differently across flight phases, and these components mediate transfer to performance. To adjudicate mechanisms and strengthen inference, future work should examine these phase and processes specific effects, along with incorporating other synchronized psychophysiology, such as EEG [for processes such as cognitive load (Borghini et al., 2014; Kyriaki et al., 2024), attention (Souza and Naves, 2021) and emotional/motivational response (Zhang et al., 2024; Liu et al., 2021)], EDA (Horvers et al., 2021), ECG/HRV (Zhou et al., 2021; Pham et al., 2021).

These findings hold practical implications for flight schools, simulator manufacturers, and instructional designers. The results point to the value of incorporating textual instructions for self-paced learning segments, possibly augmenting or replacing audio cues in certain phases (e.g., cruise navigation). For flight learning, this suggests that text-based or minimal-audio instruction may be particularly advantageous in modules requiring careful procedural focus or extended practice without real-time instructor intervention. Additionally, embedding objective and subjective measures in training modules can offer a deeper understanding of pilot states. This could enable adaptive systems that adjust the modality based on real-time workload or motivational markers. At the same time, future directions should examine how modalities impact learning in longer session, how novices and advanced learners respond differently to text-based training, how multi-crew communication factors in, and whether augmented or virtual reality solutions could amplify these benefits by merging textual feedback with head-up displays. This study helped refine knowledge on instructional modality, helping aviation stakeholders to better align simulator-based training with the cognitive, attentional, and motivational demands that define competent, confident pilot performance.

## 6 Conclusion

This study used a multidimensional approach to examine how the incorporation of flight instructions in a FS scenario affects pilots’ performance and cognitive learning states, including cognitive load, attentional strategies, and motivational and affective responses during both instructional and evaluation flight tasks. While performance measures alone failed to detect significant differences between the experimental conditions, cognitive state monitoring revealed that the unimodal-text condition was associated with significantly lower visual transition entropy compared to the unimodal-audio group during evaluation, as well as a more positive affective experience compared to the bimodal group. Although no significant differences were found across all measures, trends suggested that the text condition supported better learning outcomes, including lower implicit cognitive load, higher perceived immersion, and higher motivation. The findings highlight the detrimental effects of split-attention in high-interactivity environments, particularly in the bimodal and audio conditions, where the cognitive demands of simultaneous auditory and visual tasks overwhelmed learners. Results suggest that sensory modalities should be tailored to task complexity, with text-based instructions potentially better suited for concurrent in-flight tasks, while bimodal instructions might be more appropriate during pre-flight phases. Future research should explore how specific flight tasks, scenarios, and sensory modalities interact to influence learning and assess whether training in simulated environments effectively transfers to real-world aviation contexts through longitudinal studies.

## Data Availability

The raw data supporting the conclusions of this article will be made available by the authors, without undue reservation.

## References

[ref1] Agrawal S. Simon A. Bech S. Bærentsen K. Forchhammer S. (2020). Defining immersion: literature review and implications for research on audiovisual experiences. J. Audio Eng. Soc. 68, 404–417. doi: 10.17743/jaes.2020.0039

[ref2] Alexander A. L. Brunyé T. Sidman J. Weil S. A. (2005). From gaming to training: a review of studies on fidelity, immersion, presence, and buy-in and their effects on transfer in pc-based simulations and games, vol. 5. Arlington, VA: DARWARS Training Impact Group, 1–14.

[ref3] Allsop J. Gray R. (2014). Flying under pressure: effects of anxiety on attention and gaze behavior in aviation. J. Appl. Res. Mem. Cogn. 3, 63–71. doi: 10.1016/j.jarmac.2014.04.010

[ref4] Aragon C.R. Hearst M.A. Improving aviation safety with information visualization: a flight simulation study. in Proceedings of the SIGCHI conference on human factors in computing systems. (2005).

[ref5] Attard-Johnson J. Ó Ciardha C. Bindemann M. (2019). Comparing methods for the analysis of pupillary response. Behav. Res. Methods 51, 83–95. doi: 10.3758/s13428-018-1108-6, PMID: 30324564 PMC6420434

[ref6] Baddeley A. (1992). Working memory. Science 255, 556–559. doi: 10.1126/science.1736359 1736359

[ref7] Bailenson J. N. Yee N. Blascovich J. Beall A. C. Lundblad N. Jin M. (2008). The use of immersive virtual reality in the learning sciences: digital transformations of teachers, students, and social context. J. Learn. Sci. 17, 102–141. doi: 10.1080/10508400701793141

[ref8] Baldwin C.L. Spence C. Bliss J.P. Brill J.C. Wogalter M.S. Mayhorn C.B.. Multimodal cueing: the relative benefits of the auditory, visual, and tactile channels in complex environments. in Proceedings of the Human Factors and Ergonomics Society Annual Meeting. (2012). SAGE Publications Sage CA: Los Angeles, CA.

[ref9] Beatty J. (1982). Task-evoked pupillary responses, processing load, and the structure of processing resources. Psychol. Bull. 91, 276–292. doi: 10.1037/0033-2909.91.2.276, PMID: 7071262

[ref10] Borghini G. Astolfi L. Vecchiato G. Mattia D. Babiloni F. (2014). Measuring neurophysiological signals in aircraft pilots and car drivers for the assessment of mental workload, fatigue and drowsiness. Neurosci. Biobehav. Rev. 44, 58–75. doi: 10.1016/j.neubiorev.2012.10.003, PMID: 23116991

[ref11] Bornstein M. H. Jager J. Putnick D. L. (2013). Sampling in developmental science: situations, shortcomings, solutions, and standards. Dev. Rev. 33, 357–370. doi: 10.1016/j.dr.2013.08.003, PMID: 25580049 PMC4286359

[ref12] Brooks L. R. (1968). Spatial and verbal components of the act of recall. Can. J. Psychol. 22, 349–368. doi: 10.1037/h0082775

[ref13] Brunken R. Plass J. L. Leutner D. (2003). Direct measurement of cognitive load in multimedia learning. Educ. Psychol. 38, 53–61. doi: 10.1207/S15326985EP3801_7 12053529

[ref14] Butcher K.R. (2014). The multimedia principle. Cambridge, United Kingdom: Cambridge University Press.

[ref15] Button K. S. Ioannidis J. P. A. Mokrysz C. Nosek B. A. Flint J. Robinson E. S. J.. (2013). Power failure: why small sample size undermines the reliability of neuroscience. Nat. Rev. Neurosci. 14, 365–376. doi: 10.1038/nrn3475, PMID: 23571845

[ref16] Cairns P. (2019). Doing better statistics in human-computer interaction. Cambridge, England: Cambridge University Press.

[ref17] Callender M.N. Dornan W.A. Beckman W.S. Craig P.A. Gossett S., Transfer of skills from Microsoft flight simulator X to an aircraft. in 2009 International Symposium on Aviation Psychology. (2009).

[ref18] Charles R. L. Nixon J. (2019). Measuring mental workload using physiological measures: a systematic review. Appl. Ergon. 74, 221–232. doi: 10.1016/j.apergo.2018.08.028, PMID: 30487103

[ref19] Charlton S. G. (2002). “Measurement of cognitive states in test and evaluation” in Handbook of human factors testing and evaluation, M. S. Sanders, C. E. McCormick (eds.). Mahwah, New Jersey, USA: Lawrence Erlbaum Associates. 115–122.

[ref20] Dalgarno B. Lee M. J. (2010). What are the learning affordances of 3-D virtual environments? Br. J. Educ. Technol. 41, 10–32. doi: 10.1111/j.1467-8535.2009.01038.x

[ref21] Dede C. (2009). Immersive interfaces for engagement and learning. Science 323, 66–69. doi: 10.1126/science.1167311, PMID: 19119219

[ref22] DeLeeuw K. E. Mayer R. E. (2008). A comparison of three measures of cognitive load: evidence for separable measures of intrinsic, extraneous, and germane load. J. Educ. Psychol. 100, 223–234. doi: 10.1037/0022-0663.100.1.223

[ref23] Diaz-Piedra C. Rieiro H. Cherino A. Fuentes L. J. Catena A. di Stasi L. L. (2019). The effects of flight complexity on gaze entropy: an experimental study with fighter pilots. Appl. Ergon. 77, 92–99. doi: 10.1016/j.apergo.2019.01.012, PMID: 30832783

[ref24] Dirican A. C. Göktürk M. (2011). Psychophysiological measures of human cognitive states applied in human computer interaction. Procedia Comput. Sci. 3, 1361–1367. doi: 10.1016/j.procs.2011.01.016

[ref25] Ekman P. Friesen W. V. (1978). “Facial action coding system” in Environmental Psychology & Nonverbal Behavior. Palo Alto, California, USA: Consulting Psychologists Press.

[ref26] Ephrath A. Ephrath A. R. Tole J. R. Stephens A. T. Young L. R. Instrument scan—is it an indicator of the pilot's workload? Proceedings of the Human Factors Society Annual Meeting (1980) SAGE Publications: Los Angeles, CA 24 257–258.

[ref27] Fredricks J. A. Blumenfeld P. C. Paris A. H. (2004). School engagement: potential of the concept, state of the evidence. Rev. Educ. Res. 74, 59–109. doi: 10.3102/00346543074001059

[ref28] Ginns P. (2005). Meta-analysis of the modality effect. Learn. Instr. 15, 313–331. doi: 10.1016/j.learninstruc.2005.07.001

[ref29] Glaholt M.G., Eye tracking in the cockpit: a review of the relationships between eye movements and the aviators cognitive state. Ottawa, Ontario, Canada: National Research Council Canada (2014).

[ref30] Guay F. Vallerand R. J. Blanchard C. (2000). On the assessment of situational intrinsic and extrinsic motivation: the situational motivation scale (SIMS). Motiv. Emot. 24, 175–213. doi: 10.1023/A:1005614228250

[ref31] Hamel R. F. (1974). Female subjective and pupillary reaction to nude male and female figures. J. Psychol. 87, 171–175. doi: 10.1080/00223980.1974.9915687, PMID: 4443952

[ref32] Hart S. G. Staveland L. E. (1988). “Development of NASA-TLX (task load index): results of empirical and theoretical research” in Advances in psychology. P. A. Hancock, N. Meshkati (eds.). (Amsterdam, Netherlands: Elsevier), 139–183.

[ref33] Heitz R. P. Engle R. W. (2007). Focusing the spotlight: individual differences in visual attention control. J. Exp. Psychol. Gen. 136, 217–240. doi: 10.1037/0096-3445.136.2.217, PMID: 17500647

[ref34] Helleberg J. R. Wickens C. D. (2003). Effects of data-link modality and display redundancy on pilot performance: an attentional perspective. Int. J. Aviat. Psychol. 13, 189–210. doi: 10.1207/S15327108IJAP1303_01

[ref35] Horvers A. Tombeng N. Bosse T. Lazonder A. W. Molenaar I. (2021). Detecting emotions through electrodermal activity in learning contexts: a systematic review. Sensors 21:7869. doi: 10.3390/s21237869, PMID: 34883870 PMC8659871

[ref36] Isen A. M. (2004). “Positive affect facilitates thinking and problem solving” in Feelings and emotions: the Amsterdam symposium. A. S. R. Manstead, N. H. Frijda, A. H. Fischer (eds.). (Cambridge, UK: Cambridge University Press).

[ref37] Jennett C. Cox A. L. Cairns P. Dhoparee S. Epps A. Tijs T.. (2008). Measuring and defining the experience of immersion in games. Int. J. Human Comput. Stud. 66, 641–661. doi: 10.1016/j.ijhcs.2008.04.004

[ref38] Jeung H. J. Chandler P. Sweller J. (1997). The role of visual indicators in dual sensory mode instruction. Educ. Psychol. 17, 329–345. doi: 10.1080/0144341970170307

[ref39] Kalyuga S. (2008). Managing cognitive load in adaptive multimedia learning. Hershey, Pennsylvania, USA: IGI Global.

[ref40] Komogortsev O. V. Gobert D. V. Jayarathna S. Koh D. H. Gowda S. M. (2010). Standardization of automated analyses of oculomotor fixation and saccadic behaviors. IEEE Trans. Biomed. Eng. 57, 2635–2645. doi: 10.1109/TBME.2010.2057429, PMID: 20667803

[ref41] Krejtz K. Duchowski A. Krejtz I. Szarkowska A. Kopacz A. (2016). Discerning ambient/focal attention with coefficient K. ACM Trans. Appl. Percept. 13, 1–20. doi: 10.1145/2896452

[ref42] Kyriaki K. Koukopoulos D. Fidas C. A. (2024). A comprehensive survey of eeg preprocessing methods for cognitive load assessment. IEEE Access 12, 23466–23489. doi: 10.1109/ACCESS.2024.3360328

[ref43] Laeng B. Alnaes D. (2019). “Pupillometry” in Eye movement research. C. Klein, U. Ettinger (eds.). (Cham, Switzerland: Springer), 449–502.

[ref44] Lamontagne C. Sénécal S. Fredette M. Chen S.L. Pourchon R. Gaumont Y.. User test: how many users are needed to find the psychophysiological pain points in a journey map? in Proceedings of the 1st international conference on human interaction and emerging technologies (IHIET 2019), august 22–24, 2019, Nice, France. (2020). Cham, Switzerland: Springer.

[ref45] Lancaster J. A. Casali J. G. (2008). Investigating pilot performance using mixed-modality simulated data link. Hum. Factors 50, 183–193. doi: 10.1518/001872008X250737, PMID: 18516831

[ref46] Latorella K.A. Effects of modality on interrupted flight deck performance: implications for data link in Proceedings of the human factors and ergonomics society annual meeting. (1998). Sage Publications Sage CA: Los Angeles, CA.

[ref47] Léger P. M. Courtemanche F. Fredette M. Sénécal S. (2019). “A cloud-based lab management and analytics software for triangulated human-centered research” in Information systems and neuroscience. F. D. Davis, R. Riedl, J. vom Brocke, P.-M. Léger, A. Randolph, T. Fischer (eds.). (Cham, Switzerland: Springer), 93–99.

[ref48] Lintern G. (1991). An informational perspective on skill transfer in human-machine systems. Hum. Factors 33, 251–266. doi: 10.1177/001872089103300302, PMID: 1916844

[ref49] Liu H. Zhang Y. Li Y. Kong X. (2021). Review on emotion recognition based on electroencephalography. Front. Comput. Neurosci. 15:758212. doi: 10.3389/fncom.2021.758212 34658828 PMC8518715

[ref50] Liu D. Bhagat K. K. Gao Y. Chang T. W. Huang R. (2017). The potentials and trends of virtual reality in education: A bibliometric analysis on top research studies in the last two decades. In Virtual, augmented, and mixed realities in education. Singapore: Springer Singapore.

[ref51] Loijens L. Krips O. FaceReader methodology note. A white paper by Noldus information technology. Wageningen, The Netherlands: Noldus Information Technology. (2018).

[ref52] Lounis C. Peysakhovich V. Causse M. (2021). Visual scanning strategies in the cockpit are modulated by pilots' expertise: a flight simulator study. PLoS One 16:e0247061. doi: 10.1371/journal.pone.0247061, PMID: 33600487 PMC7891757

[ref53] Mayer R. E. (ed.) (2005). “Cognitive theory of multimedia learning” in The Cambridge handbook of multimedia learning, vol. 41, 31–48.

[ref54] Mayer R. E. (2014). Incorporating motivation into multimedia learning. Learn. Instr. 29, 171–173. doi: 10.1016/j.learninstruc.2013.04.003

[ref55] Mayer R. E. (ed.) (2024). The past, present, and future of the cognitive theory of multimedia learning. Educ. Psychol. Rev. 36:8. doi: 10.1007/s10648-023-09842-1

[ref56] Mayer R. Mayer R. E. (2005). The Cambridge handbook of multimedia learning. Cambridge, United Kingdom: Cambridge university press.

[ref57] Mayer R. E. Moreno R. (1998). A split-attention effect in multimedia learning: evidence for dual processing systems in working memory. J. Educ. Psychol. 90, 312–320. doi: 10.1037/0022-0663.90.2.312

[ref58] Mayer R. E. Pilegard C. (2005). “Principles for managing essential processing in multimedia learning: segmenting, pretraining, and modality principles” in The Cambridge handbook of multimedia learning, R. E. Mayer (editor). Cambridge, United Kingdom: Cambridge University Press. 169–182.

[ref59] McGann A. Morrow D. Rodvold M. Mackintosh M. A. (1998). Mixed-media communication on the flight deck: a comparison of voice, data link, and mixed ATC environments. Int. J. Aviat. Psychol. 8, 137–156. doi: 10.1207/s15327108ijap0802_4

[ref60] Miguel-Alonso I. Checa D. Guillen-Sanz H. Bustillo A. (2024). Evaluation of the novelty effect in immersive virtual reality learning experiences. Virtual Reality 28:27. doi: 10.1007/s10055-023-00926-5

[ref61] Moreno R. (2005). “Instructional technology: promise and pitfalls” in Technology-based education: Bringing researchers and practitioners together, M. Orey, V. J. McClendon, R. M. Branch (eds). Greenwich, Connecticut, USA: Information Age Publishing. 1–19.

[ref62] Moreno R. (2006). Does the modality principle hold for different media? A test of the method-affects-learning hypothesis. J. Comput. Assist. Learn. 22, 149–158. doi: 10.1111/j.1365-2729.2006.00170.x

[ref63] Moreno R. (2007). Optimising learning from animations by minimising cognitive load: cognitive and affective consequences of signalling and segmentation methods. Appl. Cogn. Psychol. 21, 765–781. doi: 10.1002/acp.1348

[ref64] Moreno R. (2009). Learning from animated classroom exemplars: the case for guiding student teachers' observations with metacognitive prompts. Educ. Res. Eval. 15, 487–501. doi: 10.1080/13803610903444592

[ref65] Moreno R. Mayer R. E. (1999). Cognitive principles of multimedia learning: the role of modality and contiguity. J. Educ. Psychol. 91, 358–368. doi: 10.1037/0022-0663.91.2.358

[ref66] Myers P. L. III Starr A. W. Mullins K. (2018). Flight simulator fidelity, training transfer, and the role of instructors in optimizing learning. Int. J. Aviation Aeronautics Aerospace 5:6. doi: 10.15394/ijaaa.2018.1203

[ref67] Noetel M. Griffith S. Delaney O. Harris N. R. Sanders T. Parker P.. (2022). Multimedia design for learning: an overview of reviews with meta-meta-analysis. Rev. Educ. Res. 92, 413–454. doi: 10.3102/00346543211052329

[ref68] Noldus Information Technology (2015). FaceReader (Version 6.0). Wageningen, The Netherlands: Noldus Information Technology.

[ref69] Nunnally J. C. Knott P. D. Duchnowski A. Parker R. (1967). Pupillary response as a general measure of activation. Percept. Psychophys. 2, 149–155. doi: 10.3758/BF03210310

[ref70] Olsson-Collentine A. Van Assen M. A. Hartgerink C. H. (2019). The prevalence of marginally significant results in psychology over time. Psychol. Sci. 30, 576–586. doi: 10.1177/0956797619830326, PMID: 30789796 PMC6472145

[ref71] Paivio A. (1991). Dual coding theory: retrospect and current status. Can. J. Psychol. 45, 255–287. doi: 10.1037/h0084295

[ref72] Pattemore A. Muñoz C. (2024). Perceptions of learning from audiovisual input and changes in L2 viewing preferences: the roles of on-screen text and proficiency. ReCALL 36, 135–151. doi: 10.1017/S0958344024000065

[ref73] Pham T. Lau Z. J. Chen S. A. Makowski D. (2021). Heart rate variability in psychology: a review of HRV indices and an analysis tutorial. Sensors 21:3998. doi: 10.3390/s21123998 34207927 PMC8230044

[ref74] Pociask F. D. Morrison G. (2004). The effects of Split-attention and redundancy on cognitive load when learning cognitive and psychomotor tasks. Bloomington, Indiana, USA: Association for Educational Communications and Technology.

[ref75] Rehmann A. J. (1997). Human factors recommendations for airborne controller-pilot data link communications (CPDLS) systems: a synthesis of research results and literature. Federal Aviation Administration Technical Center, Atlantic City, NJ.

[ref76] Salvucci D.D. Goldberg J.H. Identifying fixations and saccades in eye-tracking protocols. in Proceedings of the 2000 symposium on eye tracking research & applications (2000).

[ref77] SAS Institute Inc. (2013), SAS (Version 9.4). Cary, North Carolina, USA: SAS Institute Inc.

[ref78] Schneider W. Shiffrin R. M. (1977). Controlled and automatic human information processing: I. Detection, search, and attention. Psychol. Rev. 84, 1–66. doi: 10.1037/0033-295X.84.1.1

[ref79] Scott T. R. Wells W. H. Wood D. Z. (1967). Pupillary response and sexual interest reexamined. J. Clin. Psychol. 23, 433–438. doi: 10.1002/1097-4679(196710)23:4<433::AID-JCLP2270230408>3.0.CO;2-2, PMID: 5612380

[ref80] Shiferaw B. Downey L. Crewther D. (2019). A review of gaze entropy as a measure of visual scanning efficiency. Neurosci. Biobehav. Rev. 96, 353–366. doi: 10.1016/j.neubiorev.2018.12.007, PMID: 30621861

[ref81] Slater M. Wilbur S. (1997). A framework for immersive virtual environments (FIVE): speculations on the role of presence in virtual environments. Presence Teleop. Virt. Environ. 6, 603–616. doi: 10.1162/pres.1997.6.6.603

[ref82] Souza R. H. C. E. Naves E. L. M. (2021). Attention detection in virtual environments using EEG signals: a scoping review. Front. Physiol. 12:727840. doi: 10.3389/fphys.2021.727840, PMID: 34887770 PMC8650681

[ref83] Tobii Pro, Pro Lab User Manual. Sweden: Tobii Pro AB. (2021).

[ref84] Tsay C. H. H. Kofinas A. K. Trivedi S. K. Yang Y. (2020). Overcoming the novelty effect in online gamified learning systems: an empirical evaluation of student engagement and performance. J. Comput. Assist. Learn. 36, 128–146. doi: 10.1111/jcal.12385

[ref85] Unema P. J. Pannasch S. Joos M. Velichkovsky B. M. (2005). Time course of information processing during scene perception: the relationship between saccade amplitude and fixation duration. Vis. Cogn. 12, 473–494. doi: 10.1080/13506280444000409

[ref86] Valverde H. H. (1973). A review of flight simulator transfer of training studies. Hum. Factors 15, 510–522. doi: 10.1177/001872087301500603

[ref87] Van De Merwe K. Van Dijk H. Zon R. (2012). Eye movements as an indicator of situation awareness in a flight simulator experiment. Int. J. Aviat. Psychol. 22, 78–95. doi: 10.1080/10508414.2012.635129

[ref88] van der Wel P. van Steenbergen H. (2018). Pupil dilation as an index of effort in cognitive control tasks: a review. Psychon. Bull. Rev. 25, 2005–2015. doi: 10.3758/s13423-018-1432-y 29435963 PMC6267528

[ref89] van Dijk H. van de Merwe K. Zon R. (2011). A coherent impression of the pilots' situation awareness: studying relevant human factors tools. Int. J. Aviat. Psychol. 21, 343–356. doi: 10.1080/10508414.2011.606747

[ref90] Weber P. Rupprecht F. Wiesen S. Hamann B. Ebert A. Assessing cognitive load via Pupillometry, in Advances in artificial intelligence and applied cognitive computing. (2021), Cham, Switzerland: Springer. p. 1087–1096.

[ref91] Wickens C. D. Helton W. S. Hollands J. G. Banbury S. (2021). Engineering psychology and human performance. Abingdon, Oxfordshire, United Kingdom: Routledge.

[ref92] Wickens C. D. Liu Y. (1988). Codes and modalities in multiple resources: a success and a qualification. Hum. Factors 30, 599–616. doi: 10.1177/001872088803000505 3220489

[ref93] Wittrock M. C. (1989). Generative processes of comprehension. Educ. Psychol. 24, 345–376. doi: 10.1207/s15326985ep2404_2

[ref94] Wu J. Holsapple C. (2014). Imaginal and emotional experiences in pleasure-oriented IT usage: a hedonic consumption perspective. Inf. Manag. 51, 80–92. doi: 10.1016/j.im.2013.09.003

[ref95] Xbox Game Studios (2020). Microsoft Flight Simulator. Redmond, Washington, USA: Asobo Studio.

[ref96] Zhang Z. Fort J. M. Giménez Mateu L. (2024). Mini review: challenges in EEG emotion recognition. Front. Psychol. 14:1289816. doi: 10.3389/fpsyg.2023.1289816, PMID: 38239464 PMC10794660

[ref97] Zhou R. Wang C. Zhang P. Chen X. du L. Wang P.. (2021). ECG-based biometric under different psychological stress states. Comput. Methods Prog. Biomed. 202:106005. doi: 10.1016/j.cmpb.2021.106005, PMID: 33662803

